# Parathyroid vascular anatomy using intraoperative mapping angiography: the PARATLAS study

**DOI:** 10.1093/bjs/znae307

**Published:** 2025-03-11

**Authors:** Fares Benmiloud, Neil Tolley, Anne Denizot, Aimee Di Marco, Frederic Triponez

**Affiliations:** Endocrine Surgery Unit, Hôpital Européen Marseille, Marseille, France; Endocrine Surgery Service, Imperial College Healthcare NHS Trust, London, UK; Endocrine Surgery Unit, Hôpital Européen Marseille, Marseille, France; Endocrine Surgery Service, Imperial College Healthcare NHS Trust, London, UK; Endocrine Surgery Service, Imperial College Healthcare NHS Trust, Endocrine Surgery Unit, London, UK; Department of Thoracic and Endocrine Surgery, University Hospitals and Faculty of Medicine of Geneva, Geneva, Switzerland

## Abstract

**Background:**

Understanding vascular anatomy of the parathyroid glands (PG) is crucial during thyroidectomy. The aim of this study was to describe patterns and distribution of parathyroid vessels.

**Method:**

An analysis of all intraoperative mapping angiographies from indocyanine green injection and fluorescence imaging in patients undergoing thyroid surgery between February 2020 and September 2021. The parathyroid vessels were classified according to the pattern of contact with the thyroid. Data collection and analysis were carried out in accordance with MR004 reference methodology.

**Results:**

A total of 200 angiographies from 196 patients were analysed (159 female/37 male, mean(s.d.) age: 54.2 ± 13.7 years), and 320 PGs were assessed. The parathyroid vessels had no contact with the thyroid in 20 (6%; Type 0), a single-point of contact in 74 (23%; Type 1), a posterior path in 47 (15%; Type 2), a lateral path in 68 (21%; Type 3), an intrathyroidal path in 19 (6%; Type 4), and a possible medial path in 26 (8%; Type X1) cases. The course of the vessels was unclear in 36 cases (11%; Type X2). Spatial distribution analysis showed a concentration of superior parathyroid vessels around Zuckerkandl’s tubercle, whereas the distribution of the inferior parathyroid vessels was anterior and sparse.

**Conclusion:**

Intraoperative mapping angiographies help to define the main patterns of the parathyroid vessels according to their contact with the thyroid and provide surgically useful information about spatial vessel distribution.

## Teaser

Analysis of intraoperative mapping angiography helped describe the types of parathyroid vessels as well as their general distribution around the thyroid gland. This information can be used to optimize vascular preservation during thyroidectomy.

## Introduction

Knowledge of the vascular anatomy of the parathyroid glands (PGs) is essential to preserve the vitality of these during thyroidectomy. Postoperative hypoparathyroidism, mainly due to PG devascularization, occurs in 30–40% of patients after total thyroidectomy and is permanent in 5–10%. To date, rare descriptions based on cadavers^1,2^ or surgical observations^3–6^ have provided useful but heterogeneous information about the arrangement of the parathyroid vessels around the thyroid. Furthermore, there is no accessible anatomic atlas to inform surgeons about parathyroid vascularization.

Following the Fluogreen feasibility study^7^, which described the advantages and limitations of intraoperative parathyroid mapping angiography (iMAP) using indocyanine green (ICG), it was felt that a methodical analysis of parathyroid angiographies could provide useful information on PG vessel distribution.

The main objective of this study was thus to describe and classify the different patterns of parathyroid vessels visible by iMAP. The secondary objective was to analyse the global distribution of the vessels, to locate the zones in which the vessels are more vulnerable to injury during thyroid dissection.

## Methods

### Study design, setting, and participants

A total of 200 real-time angiography films (100 consecutive right-sided and 100 consecutive left-sided) taken with a fluorescent camera after ICG injection between February 2020 and September 2021 were retrospectively reviewed. All patients had undergone thyroid lobectomy or total thyroidectomy, with or without lymph node dissection, at the Hôpital Européen Marseille, by one surgeon (F.B.).

### Interventions, thyroid surgery and angiography

All surgeries were performed using surgical loops (2.5× magnification), a neuromonitoring system, a fine-bite sealing device and a fluorescence imaging system (Fluobeam LX, Fluoptics, Grenoble, France). A classic cervicotomy was performed and the strap muscles were dissociated. The anterior branches of the superior thyroid pedicle and the middle vein of the thyroid lobe were ligated. The lobe was then gently medialized, the PGs were exposed using autofluorescence, and angiography was performed.

Angiography was carried out once, on a single thyroid lobe, for all patients. ICG solution (Infracyanine Serb, France) was injected intravenously (1 ml, 2.5 mg) and the angiography film was recorded with a Fluobeam LX system. The films were reviewed in slow motion secondarily, and to construct a coherent vascular tree out of a complex image, drawings of what was observed were performed. The aim was to illustrate the shape of the vessels and their relationship to the thyroid, without measuring the distances, and to simplify the readability by representing only the first vessels entering the PGs, instead of the whole network surrounding them. Thus, it is likely that the drawings represent the arterial tree, but because current technology does not allow differentiation between arteries or veins, it was decided to use the general terms ‘pedicle’ or ‘vessel’. Drawings were made by a single surgeon (F.B.), and they were secondarily checked by four independent expert surgeons (F.T., A.D., A.D.M., N.T.), validating a good agreement between the drawings and films.

**Fig. 1:**
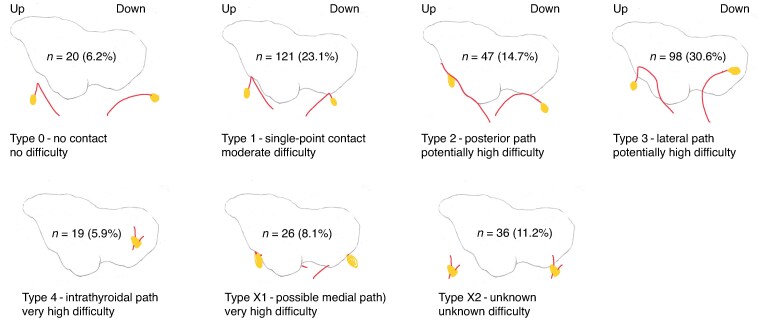
Repartition of the different vascular patterns Type 0: Parathyroid pedicle making no contact with the thyroid, with no difficulty of preservation in situ during thyroidectomy; Type 1: Parathyroid pedicle making a contact with the thyroid on a single point, moderately difficult to preserve *in situ* during thyroidectomy; Type 2: Parathyroid pedicle running along the posterior edge of the thyroid, potentially difficult to preserve *in situ* during thyroidectomy; Type 3: Parathyroid pedicle running on the lateral face of the thyroid, potentially difficult to preserve *in situ* during thyroidectomy; Type 4: Intrathyroidal parathyroid pedicle, very difficult or impossible to preserve *in situ* during thyroidectomy; Type X1: Parathyroid pedicle possibly running on the medial face of the thyroid, very difficult to preserve *in situ* during thyroidectomy and Type X2: Unknown pathway.

The preliminary drawings were then reproduced on a single thyroid model for comparison and a simplified, digitized version was produced to classify parathyroid vessels according to different patterns, and to assess the potential surgical difficulty of preserving each pattern. Type 0 vessels were distant from the thyroid and did not appear to present any difficulty in preservation (No Difficulty). Type 1 vessels had a single point of contact, <1 mm in length with the thyroid, and appeared detachable from the capsule, without having to leave any thyroid tissue in place (Moderate Difficulty). Types 2 and 3 vessels had a superficial pathway on the posterior edge and on the lateral face of the thyroid respectively, which could imply a challenging dissection, depending on their degree of fusion between the vessels and the thyroid capsule or parenchyma (Potentially High Difficulty). Type 4 vessels were intrathyroidal and considered impossible to preserve without risky intrathyroidal dissection or reimplantation (Very High Difficulty). Type X1 vascularization, in which the vascular orientation could strongly suggest parathyroid vascularization running on the medial aspect of the thyroid and hidden by it, were very difficult to preserve (Very High Difficulty), whereas it was not possible to assess the difficulty of Type X2 vascularization, in which the vascular pathway was unknown (Type X2). The types of vascular patterns are depicted in *Fig. 1*. All the drawings are displayed in *Fig. S1*.

**Fig. 2:**
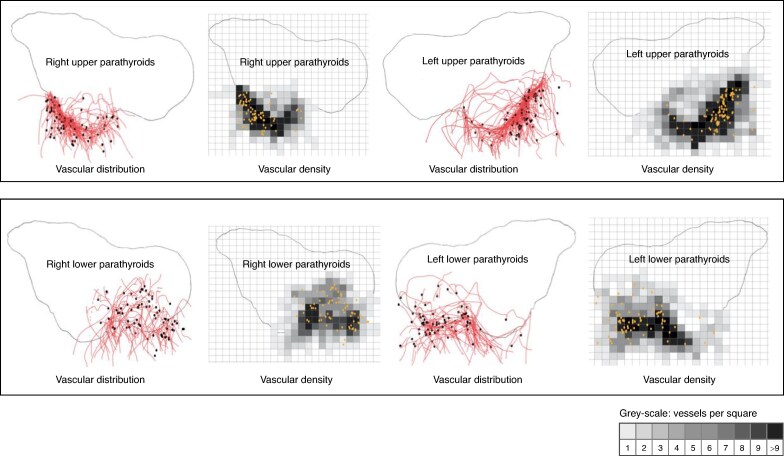
Overall distribution and density of parathyroid vessels Vascular distribution: vessels are represented by red curves and parathyroid glands by black dots. Vascular density: the grey-scale corresponds to the number of vessels counted in each scare. Orange dots correspond to the parathyroid glands.

The distribution of all parathyroid vessels around the thyroid was then represented by copying simplified digitized drawings of each of the upper and lower vessels onto the same template. The areas of vascular density were represented by dividing the space into squares, with arbitrarily chosen sizes. The vessels present in each square were then counted and assigned a grey level corresponding to the number of vessels. The whole process is depicted in *Video S1*. The overall distribution of the parathyroid vessels and density of parathyroid vascularization are represented in *Fig. 1* and the distribution of the vessels and density of vascularization according to whether the PGs were located inside or outside the thyroid area are depicted in *Fig. 2*.

### Collected variables

Other demographic and clinical data were collected and included: sex, age, indication for and extent of surgery, and occurrence of complications (hypoparathyroidism, recurrent paralysis, compressive haematoma). The quality of the information provided by angiography was classified as iMAP2 if it allowed a precise view of the parathyroid pedicle entering the PG, iMAP1 if it only gave an idea of the potential distribution of the vessels, and iMAP0 if it provided no relevant information.

### Statistical analysis

Quantitative data are reported as means ± standard deviation, and qualitative data are reported as frequency and percentage. Chi^2^ or Fisher’s tests were used for the comparison of qualitative data between groups. Multiple comparisons were assessed using the Tukey-style test. All values of *P* were considered statistically significant at α-level = 0.05. All calculations were performed using SAS version 9.4 statistical software (SAS Institute Inc., Cary, NC).

### Ethics

The study was approved by the local ethics committee and was conducted in accordance with ethical standards. Data collection and analysis followed the reference methodology MR-004 registered on No. 2022_03_01_FB in the HEM register. According to French regulations, the study was registered as reference methodology (MR-004) on the French Health Data Hub registration platform (https://www.health-data-hub.fr).

## Results

### Study participants

A total of 200 angiographies (100 consecutive right-sided and 100 consecutive left-sided) from 196 patients were analysed (159 female/37 male, mean(s.d.) age: 54.2 ± 13.7 years). Among the patients, 62 underwent lobectomy, out of which 21 were completion thyroidectomies (four patients had a lobectomy followed by a completion for cancer) and 138 underwent a total thyroidectomy (*Table 1*). Overall, 320 PGs were assessed (80% of the expected 400 PGs): 82 right upper, 73 right lower, 89 left upper, and 76 left lower. Angiography was iMAP2, iMAP1, or iMAP0 in 184 (57%), 89 (28%), and 47 (15%) PGs respectively (*Table S1*).

**Table 1:** Characteristics of the study population

Variable	
No. of patients	196
No. of operations	200
Age (years), mean ± s.d.	54.2 ± 13.7
**Sex**	
Female	159
Male	37
**Indication**	
Multinodular goitre	72 (36%)
Nodule	32 (16%)
Grave’s disease	27 (4%)
Differentiated thyroid cancer	45 (23%)
Medullary thyroid cancer	2 (1%)
Renal metastasis	1 (1%)
Reoperation for completion thyroidectomy	21 (12%)
**Extent of resection**	
Lobectomy	34 (17%)
Completion thyroidectomy	23 (12%)
One-stage total thyroidectomy	127 (64%)
Total thyroidectomy + unilateral CNLD	11 (6%)
Total thyroidectomy + bilateral CNLD	5 (3%)
Postoperative hypoparathyroidism (PTH <10 pg/ml)*	
Transient overall	8 (5%)
Transient and treated (<2 months)	3 (2%)
Permanent	0
**Other complications**	
Recurrent nerve palsy (all unilateral and transient)†	8 (2%)
Haemorrhage	0

Values shown are *n*, or *n* (%) unless stated otherwise. *The rates (%) of postoperative hypoparathyroidism (PTH <10** **pg/ml) were calculated using, as a denominator, the 166 patients who underwent total or completion thyroidectomy. †The rate (%) of recurrent nerve palsy was calculated using the 366 nerves at risk as a denominator. CNLD, central lymph node dissection; PTH, parathyroid hormone.

### Observations

The parathyroid vessels had no contact with the thyroid (Type 0), making their preservation easy, in 20/320 PGs analysed (6%), whereas there was a single point of contact with the thyroid (Type 1), making preservation more difficult, in 121 (23%). The parathyroid vessels had a course on the posterior (Type 2) or lateral (Type 3) aspect of the thyroid, making their preservation potentially difficult, in 47 (15%) and 98 (31%) cases respectively. The vessels had an intrathyroidal course (Type 4) or were located at the medial aspect of the thyroid (Type X1), making them very difficult to preserve, in 19 (6%) and 26 (8%) cases respectively, and their path remained unknown (Type X2) in 36 (11%) (*Fig. 1*).

The distribution of the patterns for the upper and lower PGs is shown in *Table 2*. Statistical comparisons showed that the vessel distribution was different between the right and left side only for the lower PGs, with significantly more Type 1 vessels in the left lower than in the right lower PGs, and more Type 3 vessels in the right lower than in the left lower PGs. In addition, there was a significant difference between the upper and lower PGs for all types of vessels.

**Table 2:** Distribution of patterns for the superior and inferior parathyroid glands

	(A) Right superior PG	(B) Left superior PG	(C) Right inferior PG	(D) Left inferior PG	A *versus* B *P*	C *versus* D *P*	(A + B) *versus* (C + D) *P*
Type 0—no contact	6 (7%)	8 (9%)	1 (1%)	5 (7%)	0.0698	0.0003*	<0.0001**
Type 1—punctiform contact	34 (42%)	20 (23%)	3 (4%)	17 (22%)			
Type 2—posterior path	11 (13%)	26 (29%)	1 (1%)	9 (12%)			
Type 3—lateral path	14 (17%)	16 (18%)	43 (59%)	25 (33%)			
Type 4—intrathyroid	1 (1%)	0 (0%)	9 (12%)	9 (12%)			
Type X1—unknown medial path	9 (11%)	13 (15%)	3 (4%)	1 (1%)			
Type X2—unknown	7 (9%)	6 (7%)	13 (18%)	10 (13%)			
Total	82	89	73	76			

*P = 0.0003 indicates a significant difference between C and D for at least one type. After two-to-one multiple comparisons (Tukey-style test for multiple comparisons), a significant difference between C and D was confirmed for Types 1 and 3. **P < 0.0001 indicates a significant difference between superior and inferior parathyroid vessels for at least one type. After two-to-one multiple comparisons (Tukey-style test for multiple comparisons), a significant difference between superior and inferior parathyroid vessels was confirmed for all types.

**Fig. 3:**
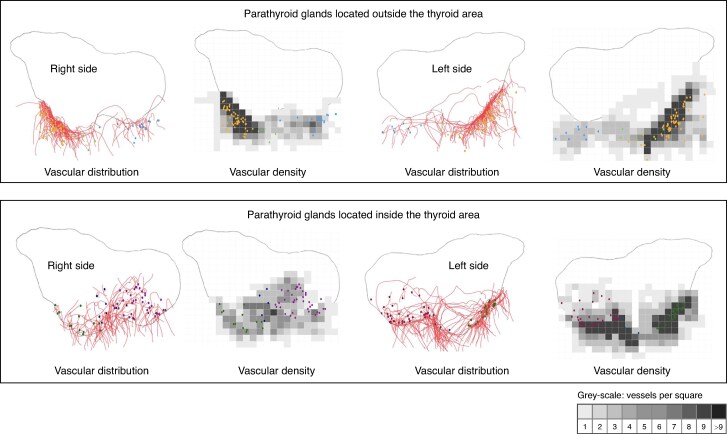
Distribution and density of parathyroid vessels according to the location of the parathyroid glands Vessels are represented by red curves and the grey-scale corresponds to the number of vessels counted in each scare. Parathyroid glands (PGs) located outside the superior (orange dot), mediolobar (light green dot), or inferior (light blue dot) part of the thyroid area. Parathyroid glands located inside the superior (dark green dot), mediolobar (dark blue dot), or inferior (purple dot) part of the thyroid area.

Schematic representation of all PGs and vessels on the same model showed that the vessels of the upper PGs tended to concentrate around the junction between the middle and upper thirds of the posterior part of the thyroid (Zuckerkandl’s tubercle zone), with a greater density at the posterior edge of the thyroid, whereas the vessels of the lower PGs had a wider dispersal area, with a relatively greater density at the posterior edge of the thyroid (*Fig. 2*). Vascularization of the PGs that were located outside the thyroid area, was most densely interposed between the PG distribution area and the posterior edge of the thyroid. This was particularly marked for PGs located opposite the upper part of Zuckerkandl’s tubercle zone (*Fig. 3*). The distribution area of the vessels feeding the PGs that were located inside the thyroid area extended laterally and anteriorly beyond the distribution area of the PGs themselves (*Fig. 3*).

### Other analyses

At the time of angiography, the PGs were already devascularized in seven cases (one right lower PG, three left upper PGs, and three left lower PGs). This devascularization was due to ligation of the vessels of the upper pole in three left upper PGs, and to ligation of the anterior vessels of the lower pole in two lower PGs (one right and one left), that were behind the branches of the pedicle. Two lower PGs were devascularized, although this could not be attributed to pedicle ligation. Both were subfascial PGs, in a mediolobar and anterior position, far from the lower pedicle (one on the right and one on the left).

Of the 159 patients who had undergone total thyroidectomy, eight (5%) had postoperative day 1 (POD1) parathyroid hormone (PTH) < 10 pg/ml, of whom three (2%) developed hypocalcaemia treated with calcium ± vitamin D, two with undetectable PTH at POD1 and one with PTH <5 pg/ml at POD1. All three patients recovered from their hypoparathyroidism within one month. There were eight recurrent paralyses, all unilateral and transient (2% of the 366 nerves at risk) and no haemorrhages (*Table 1*).

## Discussion

This study contributes new knowledge of parathyroid vessel anatomy that is directly useful to the surgeon. Moreover, it provides an iconographic atlas of parathyroid vascular anatomy. In the literature dealing with parathyroid vascularization^1–17^, only Delattre *et al*.^1^ and Flament *et al*.^2^ offer some realistic images of what the parathyroid artery pathways actually look like, with some similarities to the present angiography images. However, these do not provide a comprehensive and systematic atlas. An innovative feature of the present study is that it uses intraoperative parathyroid angiography to render images of the vessels as they appear in the surgical situation, which is an improvement in relation to findings made with the naked eye^4,5,7^ or on cadavers^1–3^.

This study also provides a new classification of parathyroid vascular patterns, with the intention to integrate all possible cases and classify them according to criteria that make sense surgically. These criteria are the presence and shape of the contact between the parathyroid pedicle and the thyroid, as these are linked to the difficulty of preserving the pedicle during thyroidectomy. In this sense, this classification is different from previous ones that define eight types of parathyroid vessels according to the main pedicle from which they originate^7^, outline five types of vessels according to their degree of fusion with the capsule and thyroid parenchyma^5^, or show six examples of vascular arrangements as a non-exhaustive illustrative figure^4^, as none of them tended to give a realistic and complete idea of possible vascular pathways.

The present classification is not intended as the end result in itself, but as a step towards an analysis of the vascular patterns and distribution areas of parathyroid vessels that would have direct surgical usefulness. For instance, the high frequency (in at least 82% of cases) of contact between parathyroid vessels and the thyroid lead to the conclusion that even when the PGs are distant from the thyroid, their pedicles are generally close, touch, or run over (and sometimes into) the thyroid. The observation that the upper parathyroid vessels generally had a ‘loop’ shape in the surgical position, with the convexity approaching or coming into contact with the thyroid in a localized area around Zuckerkandl’s tubercle, may lead the surgeon to try to identify and preserve all vascular loops touching or running along the lateral, posterior, or medial aspect of the thyroid lobe, even when the upper PGs are not visible. This can be challenging, as the parathyroid vessels are part of a complex retrolobar thyroid network of fine, tortuous, and fragile vessels, intimately connected with the recurrent nerve. Therefore, to preserve the superior parathyroid vascularization, it may be necessary to perform a near-total thyroidectomy by leaving the posterior part of Zuckerkandl’s tubercle in place, a manoeuvre popularized by Triponez and colleagues after the work of Delattre *et al*.^11,18^.

For the lower PGs, the practical impact of the present results on the dissection technique may be more modest. When the lower PGs are visible on the surface of the thyroid, the difficulty in identifying and preserving their vascularity will depend on the degree of fusion with the thyroid^5^. Even when PGs are remote from the thyroid, the vascular loops coming from the posterior cervical plane and having an anterior convexity in contact with the thyroid should be preserved, as they can be parathyroid pedicles.

Another finding that may have a direct technical influence is that, in some cases, the PGs were already devascularized at the time of the first injection, indicating that vessels had been cut early on. The surgeon should take care to identify branches of the upper thyroid pole vessels that form a loop to run downwards and backwards, which could vascularize the upper PGs, especially on the left side. Also, the PGs posterior to the vessels of the lower thyroid pole should be looked for before dividing vessels. Finally, the present data suggest, as mentioned by Wang *et al*.^6^, that the vessels of the thyrothymic PGs may originate from the mediastinum more readily than from a branch of the inferior thyroid artery, which is interesting in relation to a central lymph node dissection. However, this needs to be confirmed and quantified by a specific study, because in this area, visualization may be impaired by fat and thymic remnants.

This study is based on the analysis of objective data, namely angiograms, with interpretation and graphic representation validated by peers. Furthermore, the validity of the approach was also reflected clinically, as the rate of transient hypoparathyroidism was low and the rate of permanent hypoparathyroidism was zero. This is probably the consequence of a better knowledge of the parathyroid vascular anatomy, as well as careful attention paid to the vessels combined with an attempt to read the vascular pathways, fine vascular dissection down to the capsule level, vascular ultra-ligatures, use of fine instruments, and surgical magnifiers. The use of autofluorescence to better focus attention, anticipate parathyroid identification, and prepare the dissection, combined with intraoperative use of ICG to better guide dissection, also contributed to these results, as demonstrated previously^19–24^.

A limitation of this study is that the drawings were made by hand. This may have induced an approximation in the representation, but on the other hand, drawing the angiograms enabled making vascular trees appear when a linear reading would only allow visualization of discontinuous and fleetingly appearing sections of vessels, often hidden by adjacent structures and/or by the contrast taken up by the tissues. This would have been much more complex to perform by a digital analysis tool, which does not yet exist for this purpose. Care has been taken to maintain the proportions between the shape of the thyroid, the location of the PGs, and the path of the vessels, in relation to simple landmarks, such as the poles, the concavities, and the convexities of the thyroid. The fidelity of the proportions, the concordance between the drawing and the information provided by the angiography, as well as the surgical relevance of the information provided, was thoroughly checked by four independent experts in the field. Another limit is that, due to the design of the study, there is no information on the number of cases in which all four glands were difficult to preserve. Delattre *et al*. and Flament *et al*. found that in 5% all four PGs were at extreme risk of devascularization^1,2^, which they reconciled with the current literature incidence of permanent hypoparathyroidism. The low rates of hypoparathyroidism in the present study, however, suggest that this rate is probably overestimated. Finally, certain patterns had to be considered as ‘potentially difficult to preserve’ because the study was based on a retrospective analysis of angiographic images. To clarify this concept of preservation difficulty, a prospective multicentre study linking the angiographic image, the assessment of what was seen and, ideally, the state of parathyroid perfusion at the end of dissection, would be an interesting follow-up to this study.

## Supplementary Material

znae307_Supplementary_Data

## Data Availability

Data are available for sharing on request to the corresponding author.
