# Supplementary material for: Parathyroid vascular anatomy using intraoperative mapping angiography: the PARATLAS study
Source: Br J Surg. 2025 Mar 11;112(3):znae307. doi: 10.1093/bjs/znae307 (PMC11894526; doi:10.1093/bjs/znae307)
Supplement: znae307_Supplementary_Data [file znae307_supplementary_data.zip › Supplemental_Figure_1.pdf]

Comprehensive and systematic atlas of the path of the parathyroid vessels in relation to the thyroid, based on intraoperative angiographies performed on 200 patients.

Introduction / Methods

This document details simplified and standardized drawings of the superior and inferior parathyroid main pedicles, extracted from intraoperative fluorescence-based angiographies performed on 200 patients. The drawings were classified according to 6 different types of patterns of parathyroid vascularization: Type 0 when the parathyroid pedicle made no contact with the thyroid, Type 1 when the parathyroid main pedicle made a punctiform contact with the thyroid, Type 2 when the parathyroid main pedicle ran along the posterior edge of the thyroid, Type 3 when the parathyroid main pedicle ran on the lateral face of the thyroid, Type 4 when the parathyroid main pedicle was intrathyroid, Type X1 when the parathyroid pedicle possibly ran on the medial face of the thyroid, and Type X2 when the course of the pedicle(s) of the parathyroid was not visible.

Each drawing is numbered as follows: Right or Left (R or L), Superior or Inferior (S or I) Parathyroid gland- Pattern Type 0, 1, 2, 3, 4, X1 or X2 and number of the drawing. Ex: RI-2.4 is the drawing # 4 for the Right Inferior Parathyroid Glands which have a Pattern Type 2 vascularization.

For each drawing, the quality of the angiography (iMAP for intraoperative mapping angiography of the parathyroid glands) from which the drawing was made, is notified: iMAP2 if it allowed a precise view of the parathyroid pedicle entering the parathyroid, iMAP1 if it only gave an idea of the potential distribution of the vessels, and iMAP0 if it provided no relevant information.

Table of content

Superior parathyroid glands : page 2
Type 0 - parathyroid pedicle making no contact with the thyroid: page 3
Type 1 - parathyroid pedicle making punctiform contact with the thyroid: page 4 - 10
Type 2 - parathyroid pedicle running along the posterior edge of the thyroid: page 11-14
Type 3 - parathyroid pedicle running on the lateral face of the thyroid: page 16-19
Type 4 - intrathyroid parathyroid pedicle: page 20
Type X1 - parathyroid pedicle possibly running on the medial face of the thyroid: page 21-23
Type X2 - unkown: page 23-25

Inferior parathyroid glands :page 26
Type 0 - parathyroid pedicle making no contact with the thyroid: page 27-28
Type 1 - parathyroid pedicle making punctiform contact with the thyroid: page 28-31
Type 2 - parathyroid pedicle running along the posterior edge of the thyroid: page 31-32
Type 3 - parathyroid pedicle running on the lateral face of the thyroid: page 32-41
Type 4 - intrathyroid parathyroid pedicle: page 42-43
Type X1 - parathyroid pedicle possibly running on the medial face of the thyroid: page 43-44
Type X2 - unkown: page 44-47

Superior Parathyroid Glands

Pattern Type 0 - Parathyroid pedicle making no contact with the thyroid
Right Side - Superior Parathyroid Glands

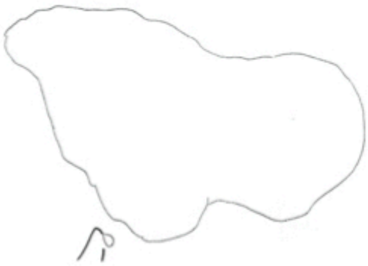

RS-0.1 iMAP2

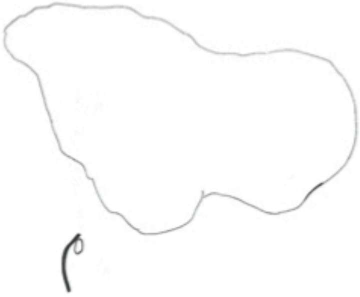

RS-0.2 iMAP2

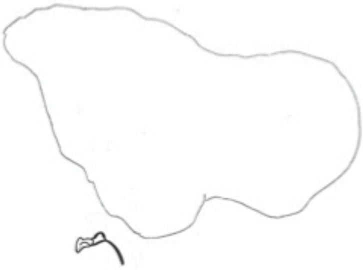

RS-0.3 iMAP2

Type 0 - Parathyroid pedicle making no contact with the thyroid
Left Side- Superior Parathyroid Glands

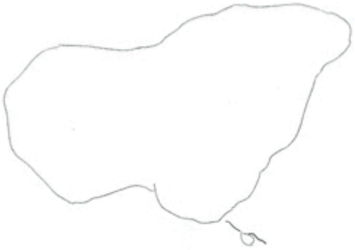

LS-0.1 iMAP0

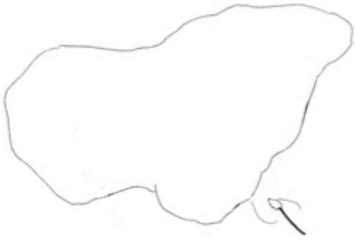

LS-0.2 iMAP2

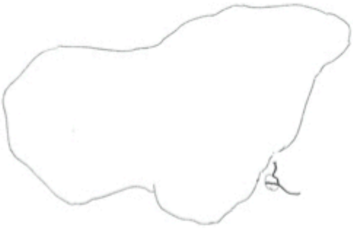

LS-0.3 iMAP2

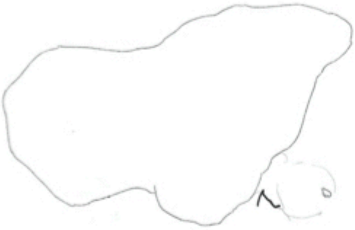

LS-0.4 iMAP0

Pattern Type 1 - Parathyroid pedicle making punctiform contact with the thyroid
Right Side - Superior Parathyroid Glands

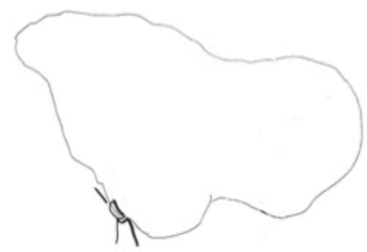

RS-1.1 iMAP2

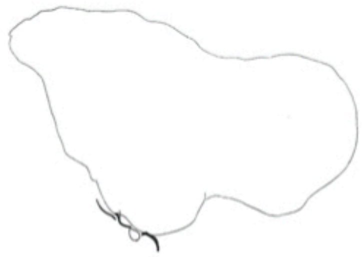

RS-1.2 iMAP2

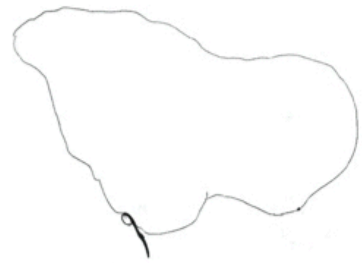

RS-1.3 iMAP2

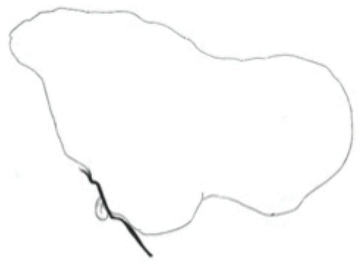

RS-1.4 iMAP2

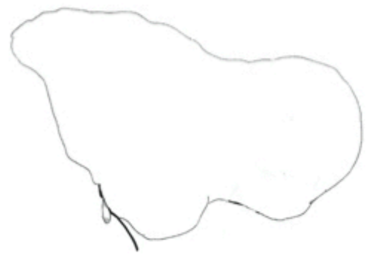

RS-1.5 iMAP2

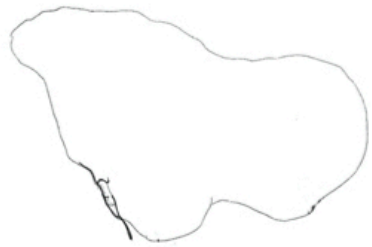

RS-1.6 iMAP2

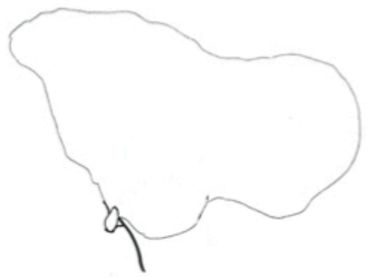

RS-1.7 iMAP2

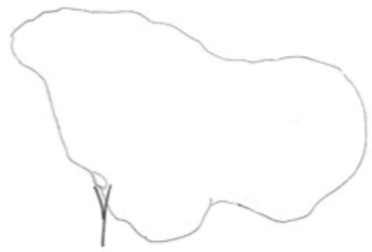

RS-1.8 iMAP1

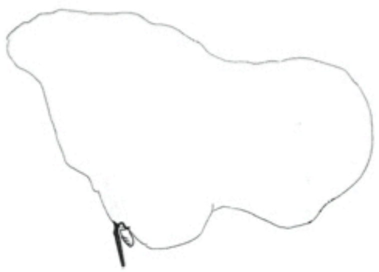

RS-1.9 *iMAP2*

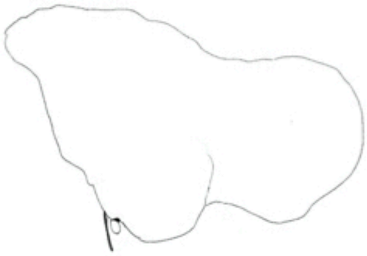

RS-1.10 *iMAP1*

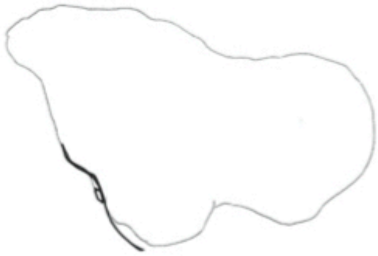

RS-1.11 *iMAP1*

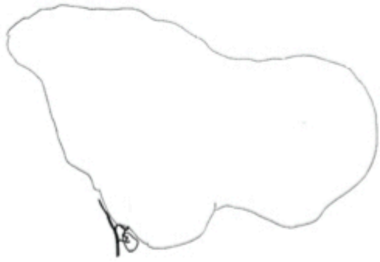

RS-1.12 *iMAP2*

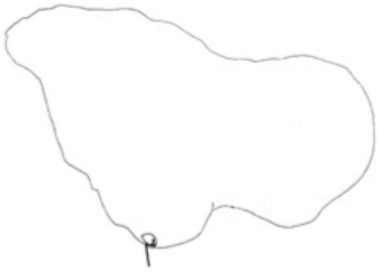

RS-1.13 *iMAP2*

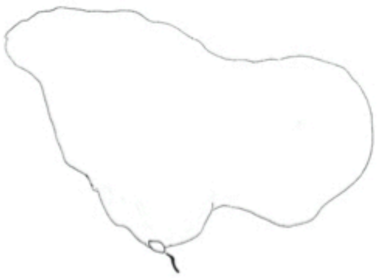

RS-1.14 *iMAP1*

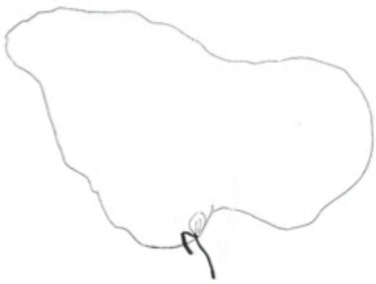

RS-1.15 *iMAP2*

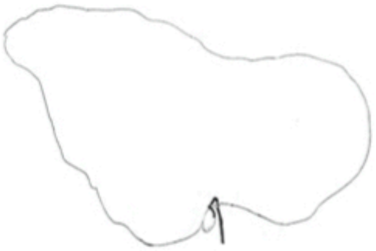

RS-1.16 *iMAP2*

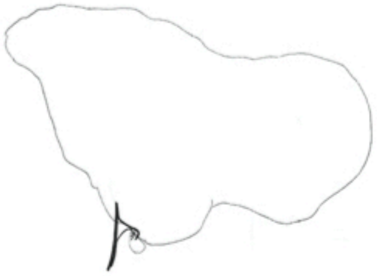

RS-1.17 *iMAP2*

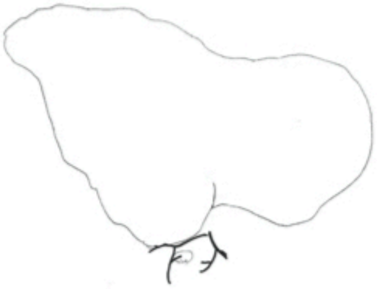

RS-1.18 *iMAP1*

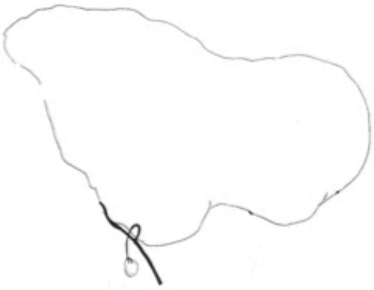

RS-1.19 *iMAP2*

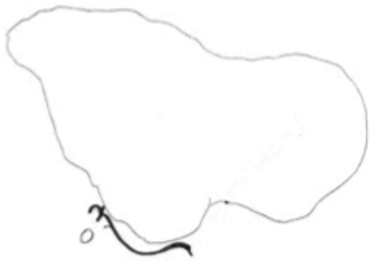

RS-1.20 *iMAP1*

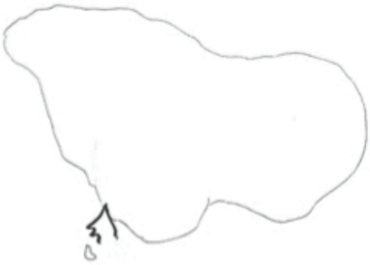

RS-1.21 *iMAP1*

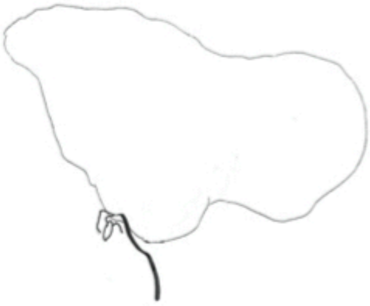

RS-1.22 *iMAP2*

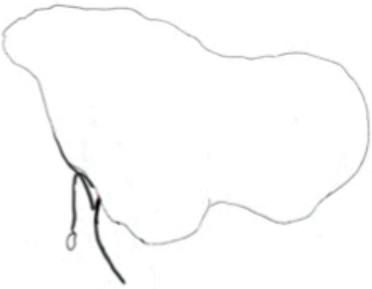

RS-1.23 *iMAP2*

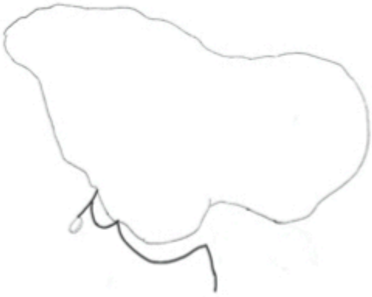

RS-1.24 *iMAP2*

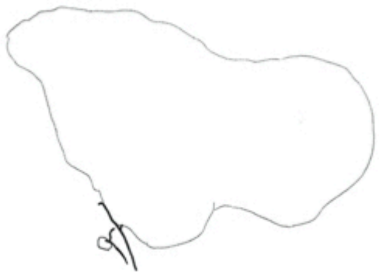

RS-1.25 *iMAP2*

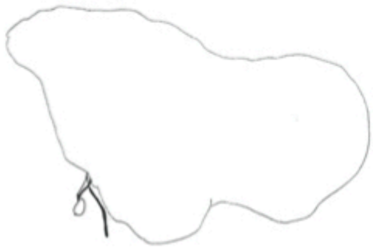

RS-1.26 *iMAP2*

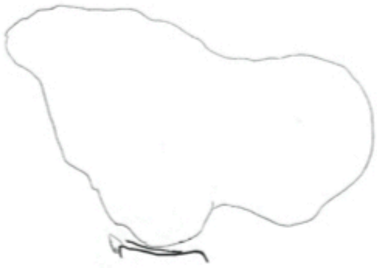

RS-1.27 *iMAP2*

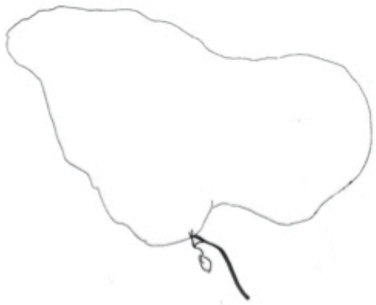

RS-1.28 *iMAP2*

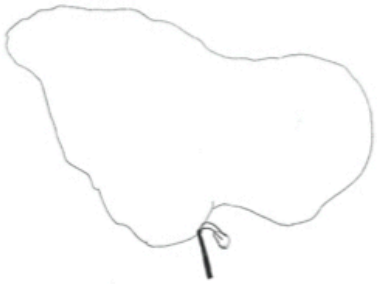

RS-1.29 *iMAP2*

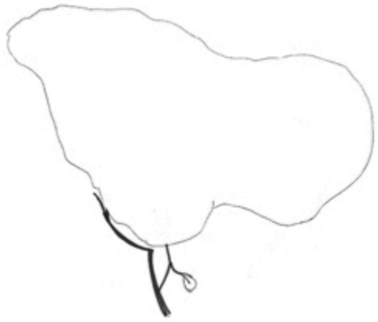

RS-1.30 *iMAP2*

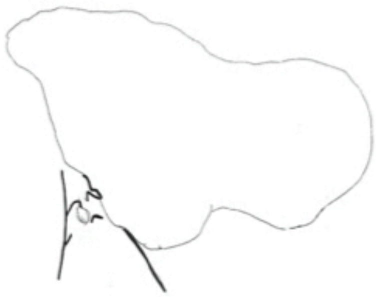

RS-1.31 *iMAP2*

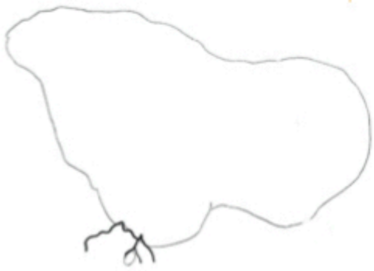

RS-1.32 *iMAP2*

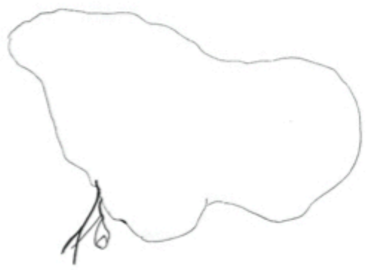

RS-1.33 iMAP2

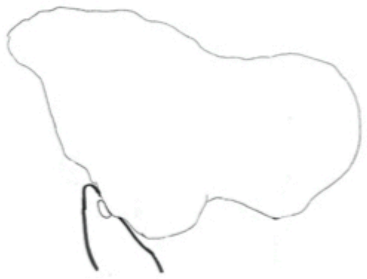

RS-1.34 iMAP1

Pattern Type 1 - Parathyroid pedicle making punctiform contact with the thyroid
Left Side - Superior Parathyroids

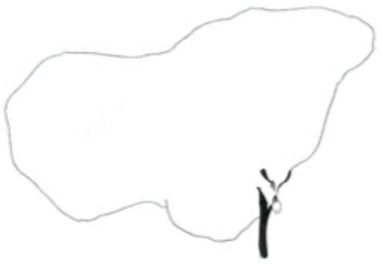

LS-1.1 iMAP1

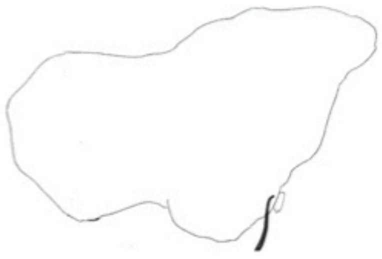

LS-1.2 iMAP0

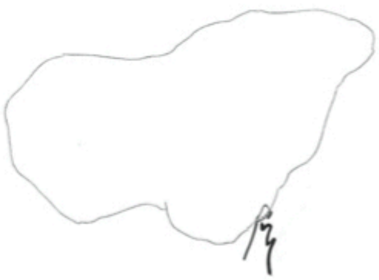

LS-1.3 iMAP1

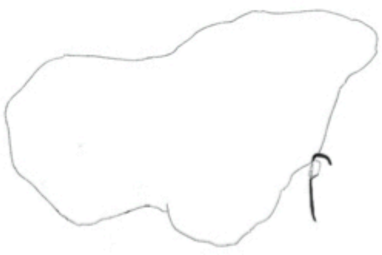

LS-1.4 iMAP1

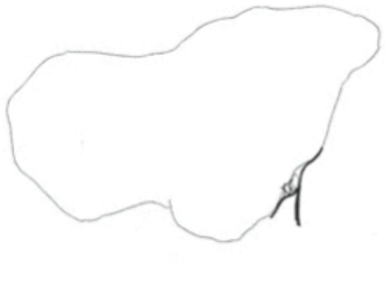

LS-1.5 *iMAP2*

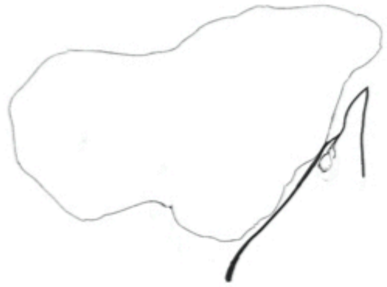

LS-1.6 *iMAP2*

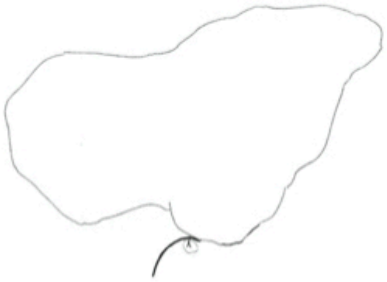

LS-1.7 *iMAP2*

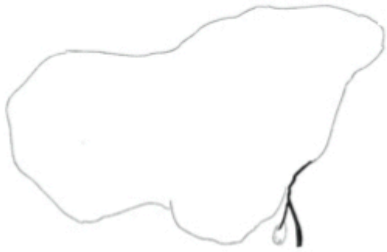

LS-1.8 *iMAP2*

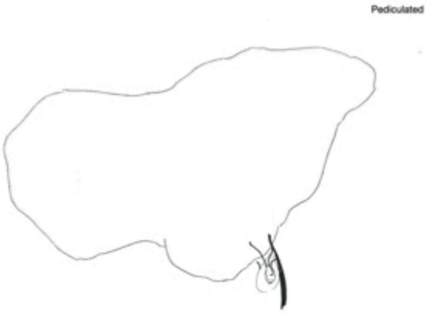

LS-1.9 *iMAP2*

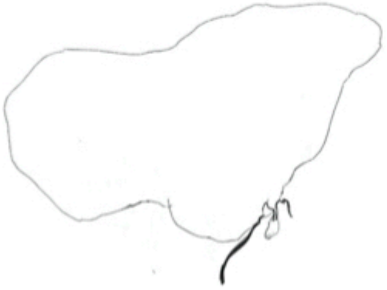

LS-1.10 *iMAP2*

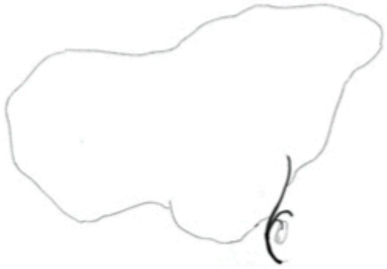

LS-1.11 *iMAP2*

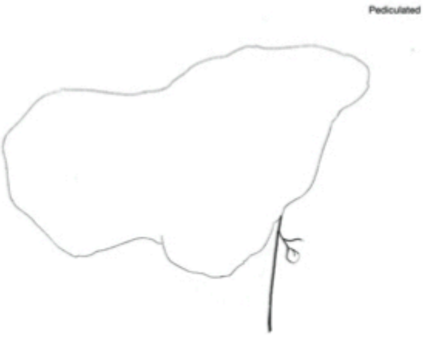

LS-1.12 *iMAP2*

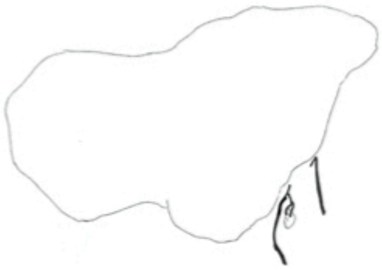

LS-1.13 *iMAP2*

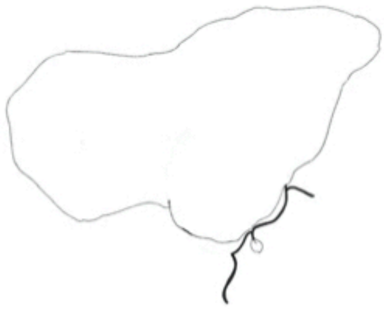

LS-1.14 *iMAP2*

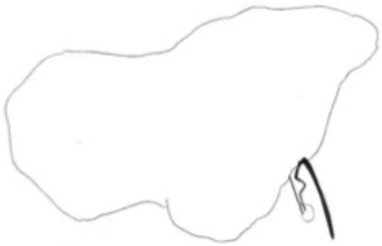

LS-1.15 *iMAP2*

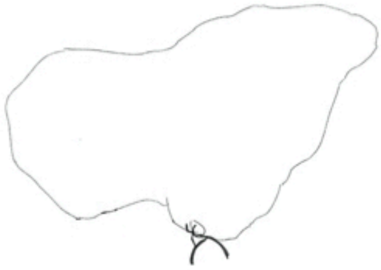

LS-1.16 *iMAP2*

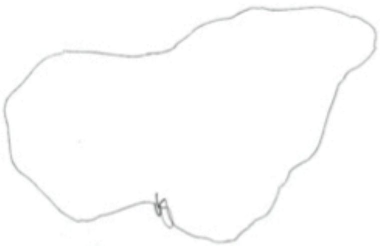

LS-1.17 *iMAP0*

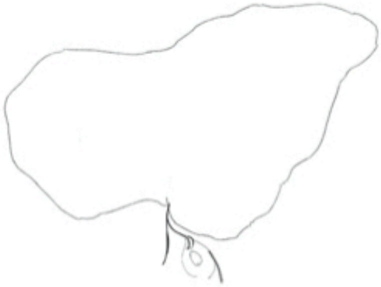

LS-1.18 *iMAP1*

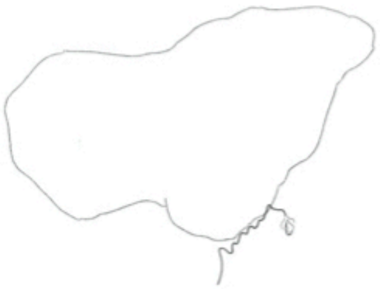

LS-1.19 *iMAP2*

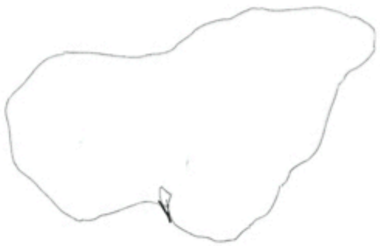

LS-1.20 *iMAP1*

Type 2 - Parathyroid pedicle running along the posterior edge of the thyroid
Right Side - Superior Parathyroid Glands

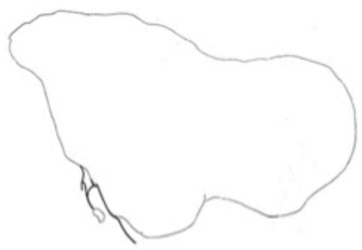

RS-2.1 iMAP2

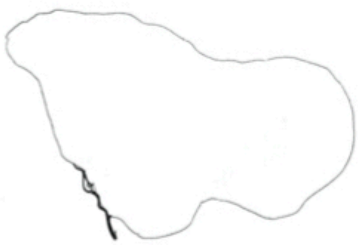

RS-2.2 iMAP2

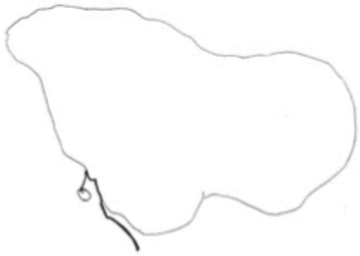

RS-2.3 iMAP2

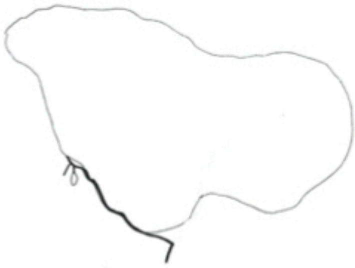

RS-2.4 iMAP2

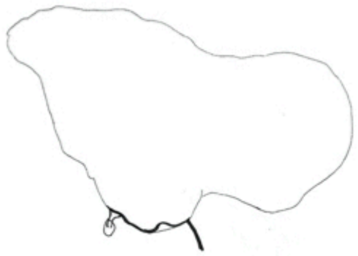

RS-2.5 iMAP2

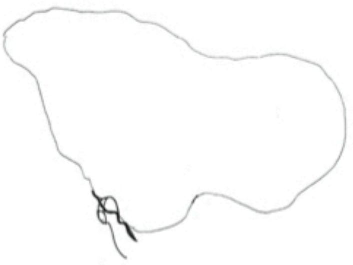

RS-2.6 iMAP2

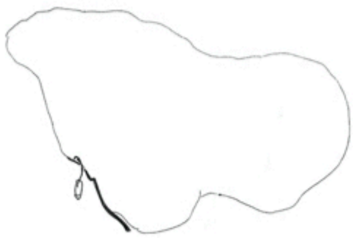

RS-2.7 iMAP2

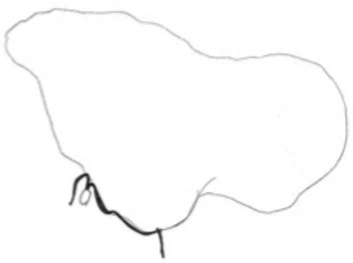

RS-2.8 iMAP2

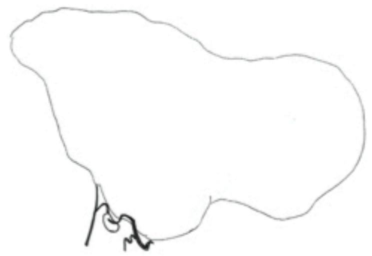

RS-2.9 iMAP2

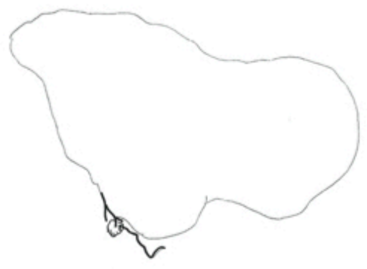

RS-2.10 iMAP1

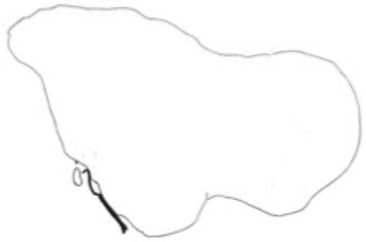

RS-2.11 iMAP1

Type 2 - Parathyroid pedicle running along the posterior edge of the thyroid
Left Side - Superior Parathyroid Glands

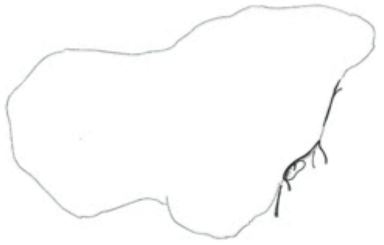

LS-2.1 iMAP2

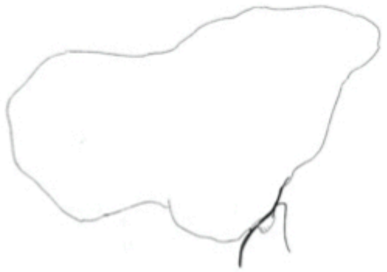

LS-2.2 iMAP2

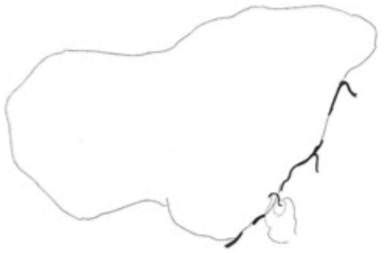

LS-2.3 iMAP1

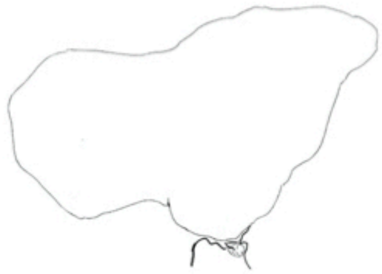

LS-2.4 iMAP2

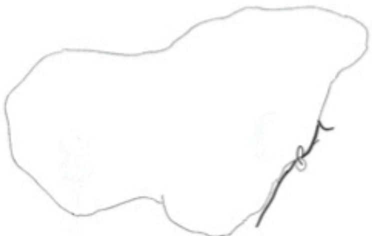

LS-2.5 *iMAP2*

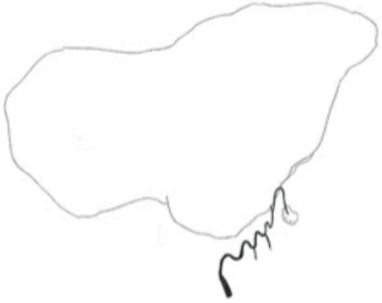

LS-2.6 *iMAP2*

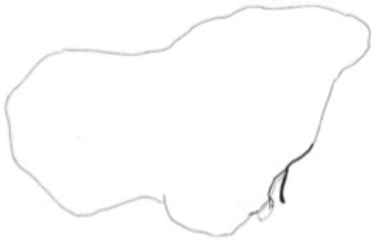

LS-2.7 *iMAP1*

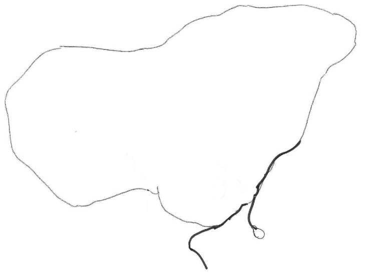

LS-2.8 *iMAP2*

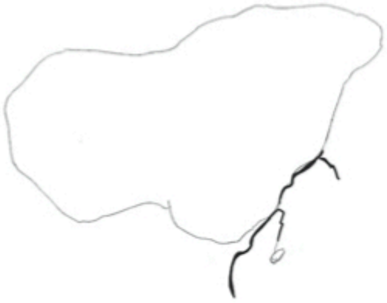

LS-2.9 *iMAP2*

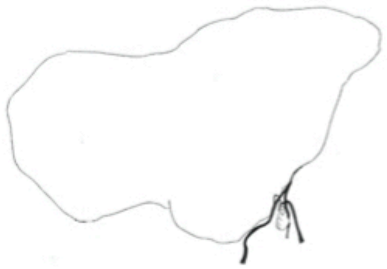

LS-2.10 *iMAP2*

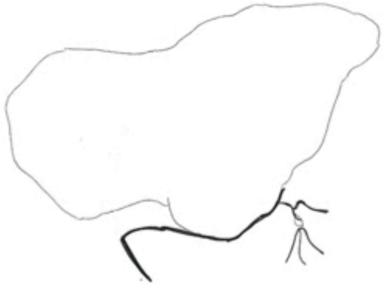

LS-2.11 *iMAP2*

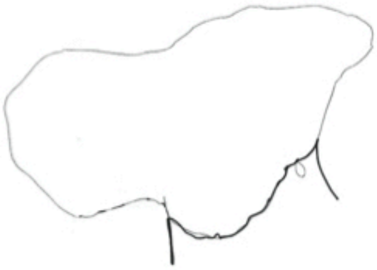

LS-2.12 *iMAP2*

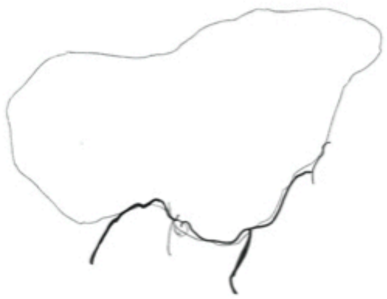

LS-2.13 *iMAP2*

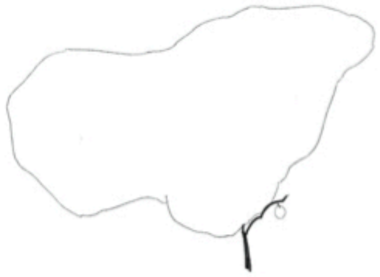

LS-2.14 *iMAP1*

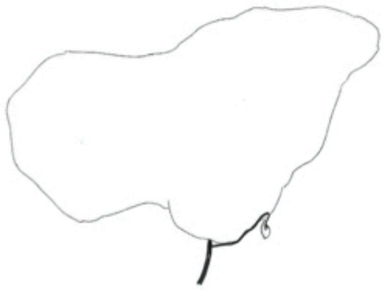

LS-2.15 *iMAP2*

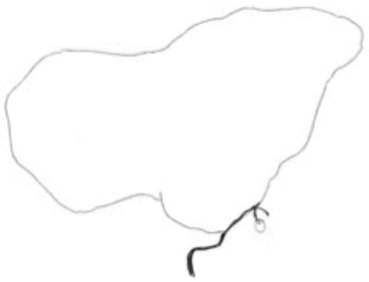

LS-2.16 *iMAP2*

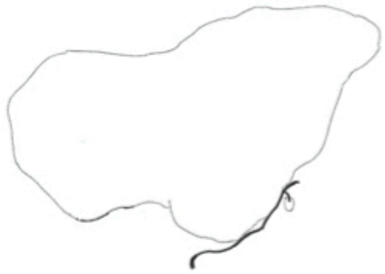

LS-2.17 *iMAP2*

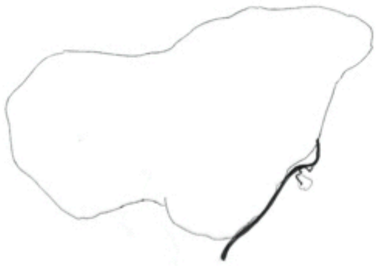

LS-2.18 *iMAP2*

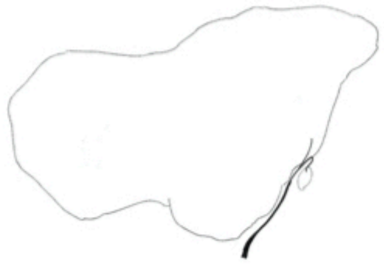

LS-2.19 *iMAP2*

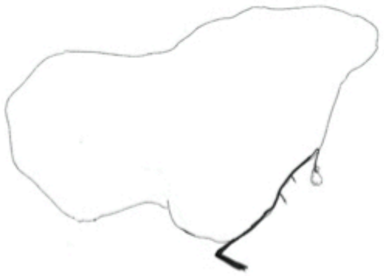

LS-2.20 *iMAP2*

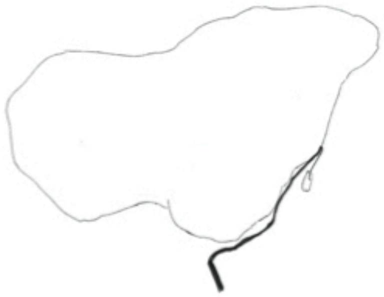

LS-2.21 *iMAP2*

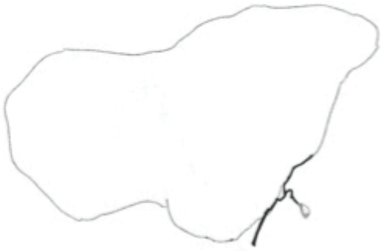

LS-2.22 *iMAP2*

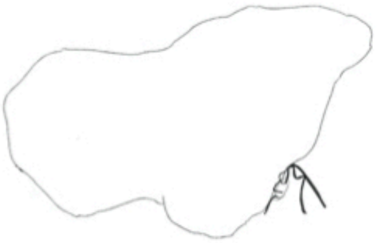

LS-2.23 *iMAP2*

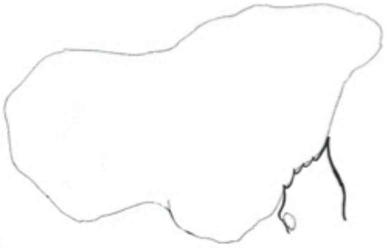

LS-2.24 *iMAP2*

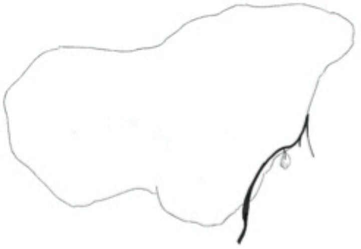

LS-2.25 *iMAP2*

Type 3 - Parathyroid pedicle running on the lateral face of the thyroid
Right Side - Superior Parathyroid Glands

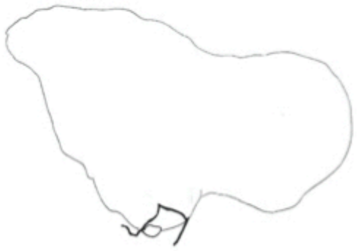

RS-3.1 iMAP2

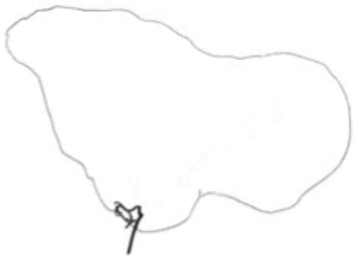

RS-3.2 iMAP2

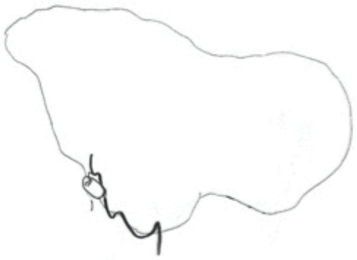

RS-3.3 iMAP2

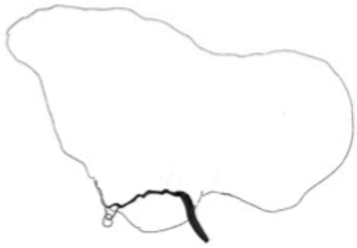

RS-3.4 iMAP2

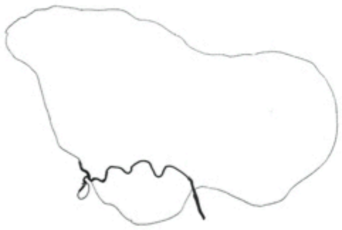

RS-3.5 iMAP2

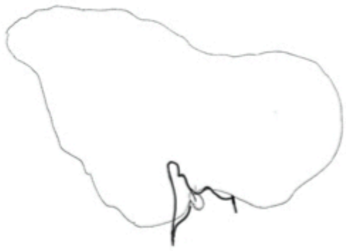

RS-3.6 iMAP2

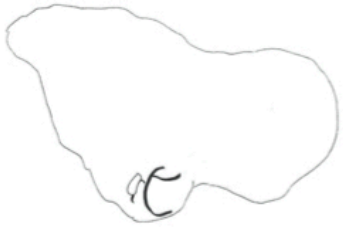

RS-3.7 iMAP2

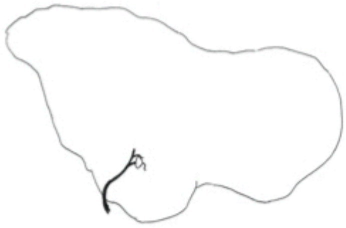

RS-3.8 iMAP2

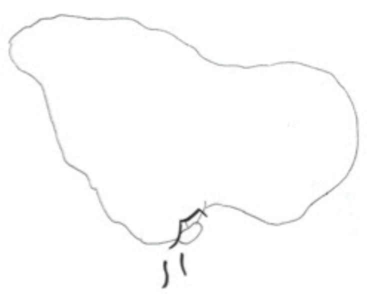

RS-3.9 *iMAP1*

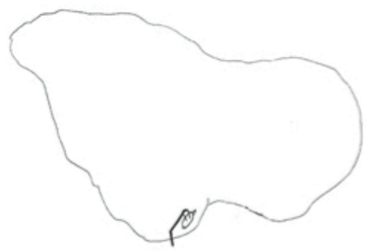

RS-3.10 *iMAP1*

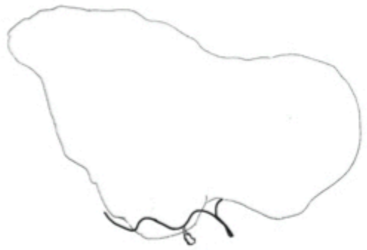

RS-3.11 *iMAP2*

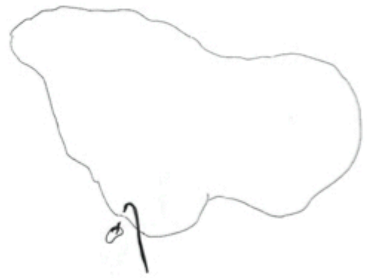

RS-3.12 *iMAP2*

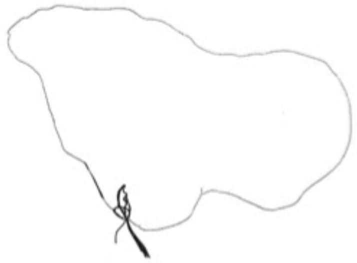

RS-3.13 *iMAP2*

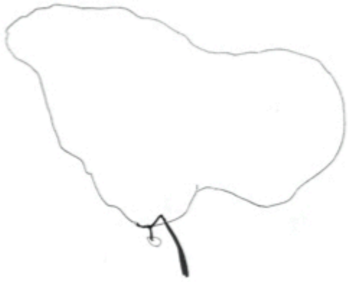

RS-3.14 *iMAP2*

Type 3 - Parathyroid pedicle running on the lateral face of the thyroid
Left Side - Superior Parathyroid Glands

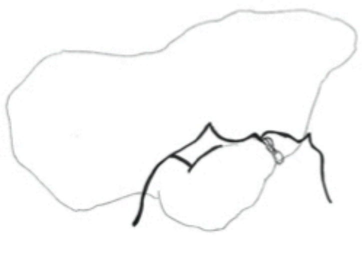

LS-3.1 *iMAP2*

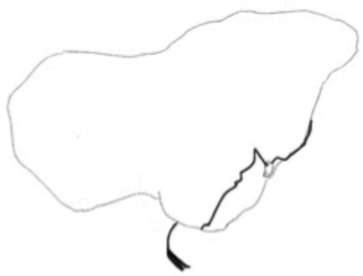

LS-3.2 *iMAP1*

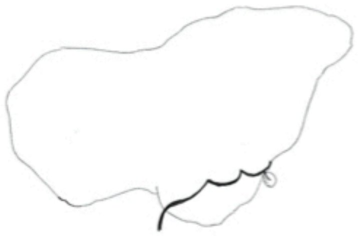

LS-3.3 *iMAP2*

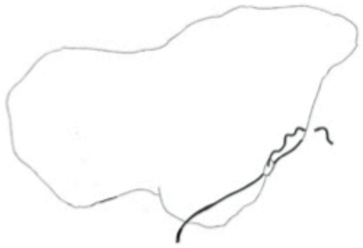

LS-3.4 *iMAP2*

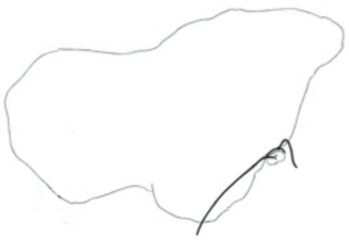

LS-3.5 *iMAP2*

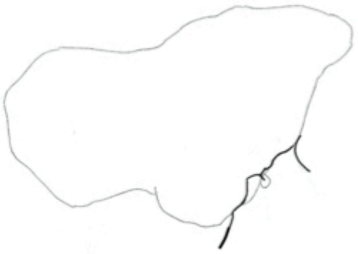

LS-3.6 *iMAP2*

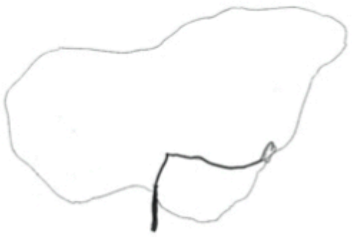

LS-3.7 *iMAP2*

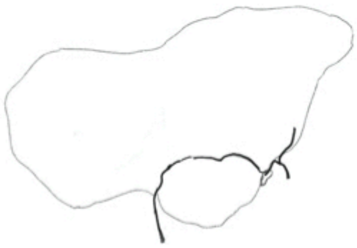

LS-3.8 *iMAP2*

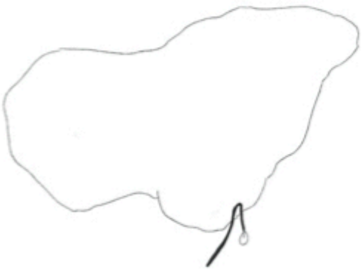

LS-3.9 *iMAP2*

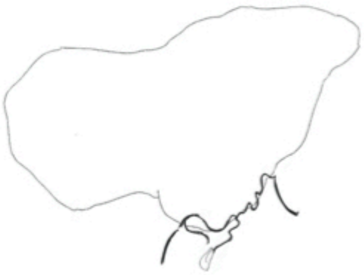

LS-3.10 *iMAP2*

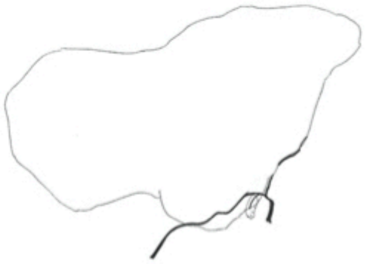

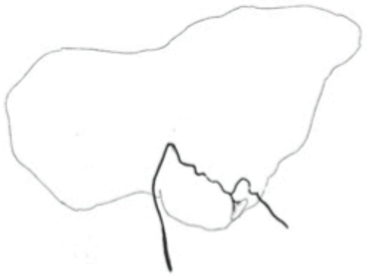

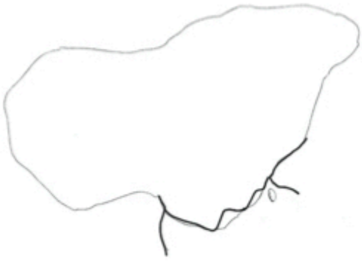

LS-3.13 *iMAP1*

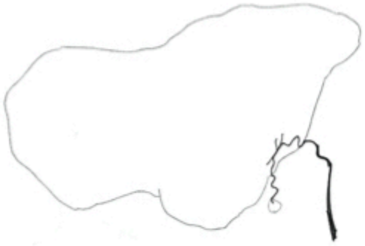

LS-3.14 *iMAP2*

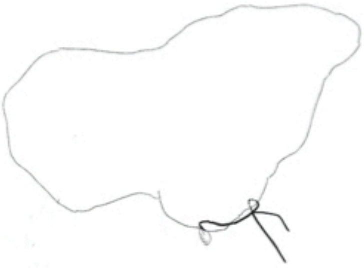

LS-3.15 *iMAP2*

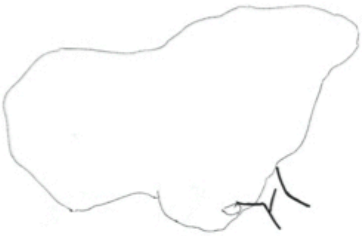

LS-3.16 *iMAP1*

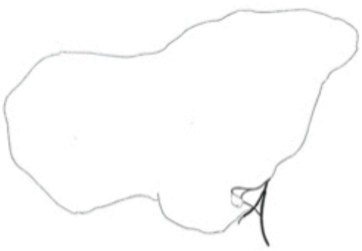

LS-3.17 iMAP2

Type 4 - Intrathyroid parathyroid
Right Side - Superior Parathyroid Glands

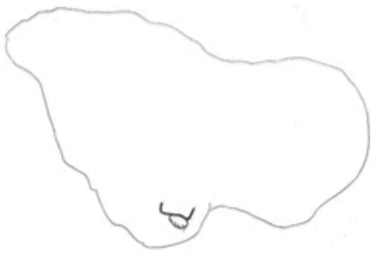

RS-4.1 iMAP0

Type X1 - Parathyroid pedicle possibly running on the medial face of the thyroid
Right Side - Superior Parathyroid Glands

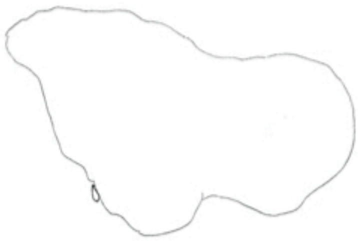

RS-X1.1 iMAP0

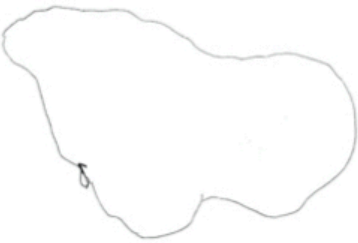

RS-X1.2 iMAP0

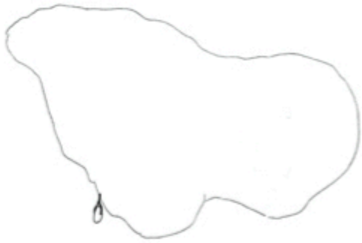

RS-X1.3 iMAP0

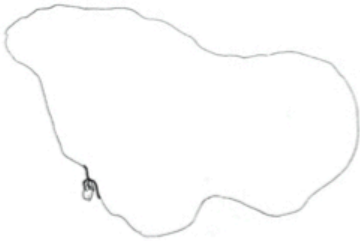

RS-X1.4 iMAP2

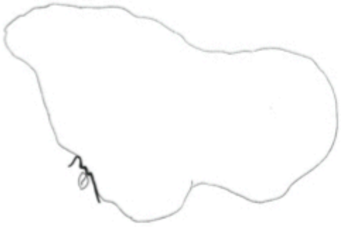

RS-X1.5 iMAP1

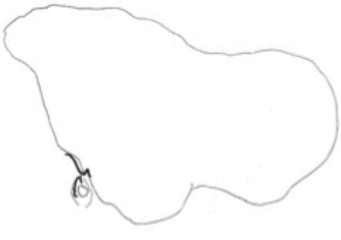

RS-X1.6 iMAP1

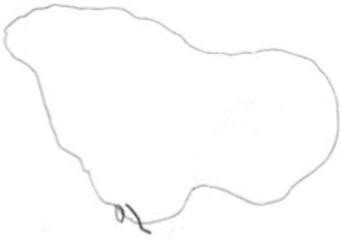

RS-X1.7 iMAP0

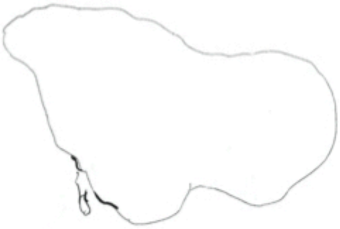

RS-X1.8 iMAP0

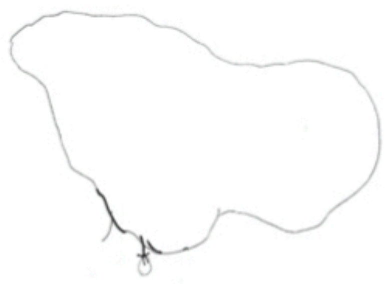

RS-X1.9 iMAP0

Type X1 - Parathyroid pedicle possibly running on the medial face of the thyroid
Left Side - Superior Parathyroid Glands

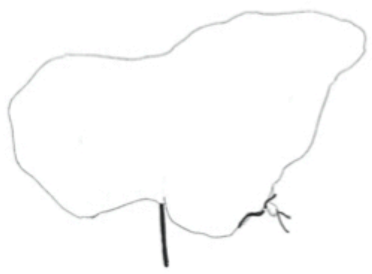

LS-X1.1 iMAP1

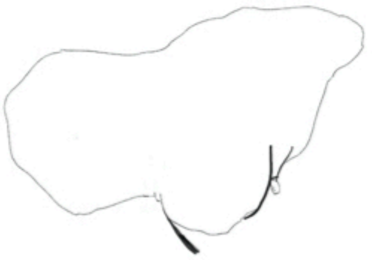

LS-X1.2 iMAP1

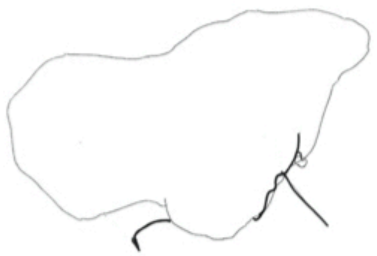

LS-X1.3 iMAP1

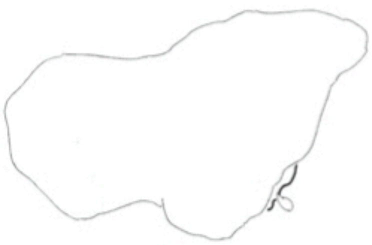

LS-X1.4 iMAP0

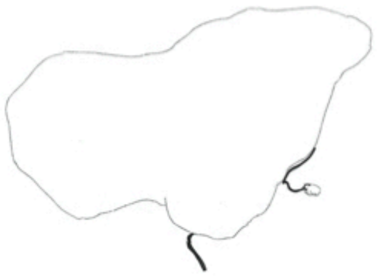

LS-X1.5 *iMAP1*

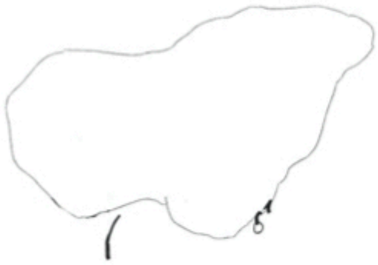

LS-X1.6 *iMAP0*

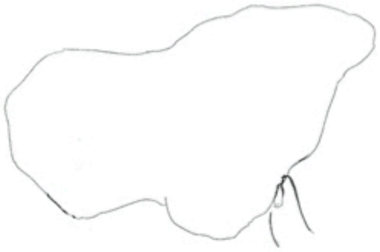

LS-X1.7 *iMAP0*

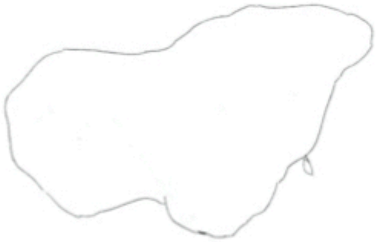

Devasc

LS-X1.8 *iMAP0*

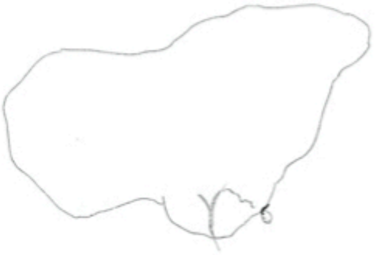

LS-X1.9 *iMAP1*

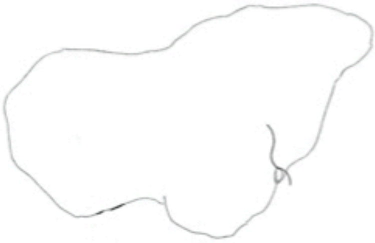

LS-X1.10 *iMAP0*

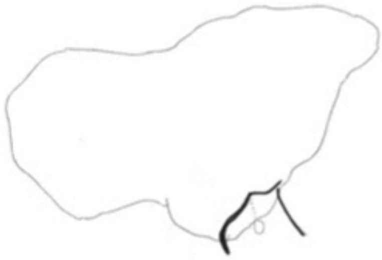

LS-X1.11 *iMAP1*

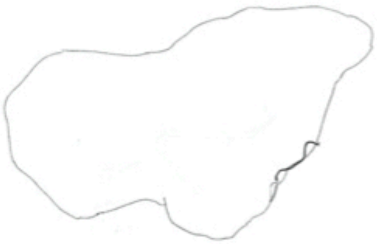

LS-X1.12 *iMAP0*

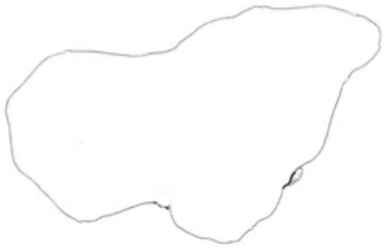

LS-X1.13 *iMAP0*

Type X2 - Parathyroid pedicle path unknown
Right Side - Superior Parathyroid Glands

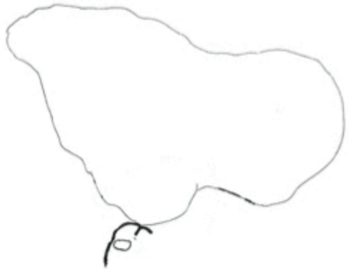

RS-X2.1 *iMAP1*

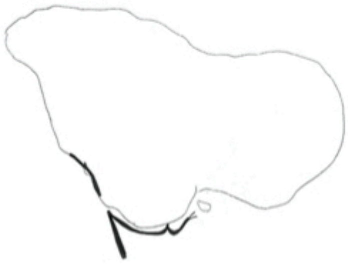

RS-X2.2 *iMAP1*

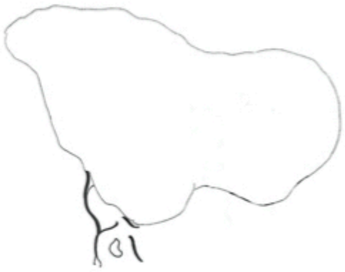

RS-X2 .3 *iMAP1*

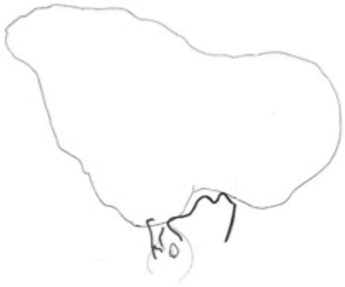

RS-X2.4 *iMAP1*

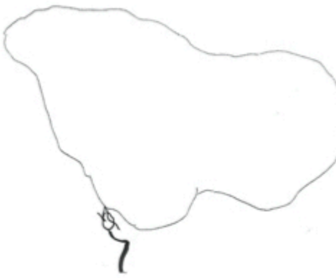

RS-X2.5 *iMAP1*

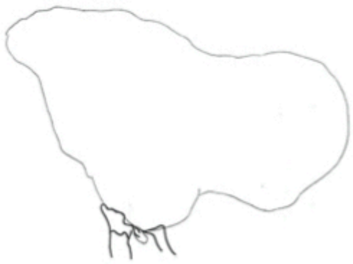

RS-X2.6 *iMAP2*

Type X2 - Parathyroid pedicle path unknown
Left Side - Superior Parathyroid Glands

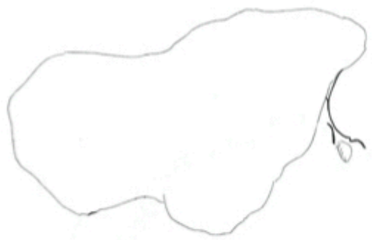

LS-X2.1 iMAP1

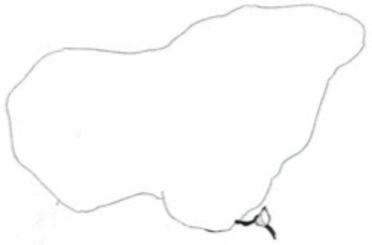

LS-X2.2 iMAP2

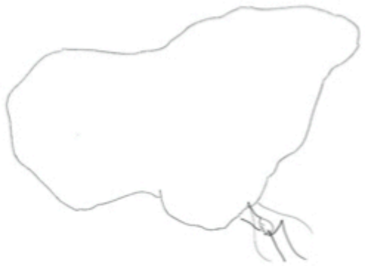

LS-X2.3 iMAP2

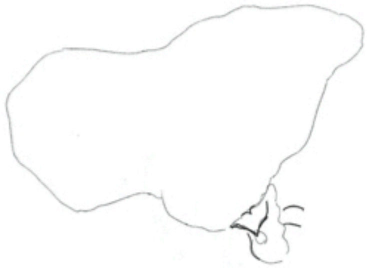

LS-X2.4 iMAP2

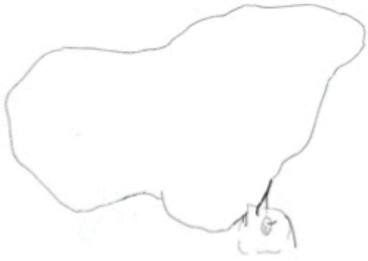

LS-X2.5 iMAP1

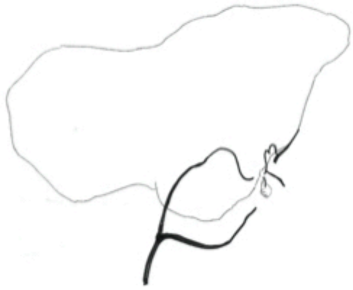

LS-X2.6 iMAP1

Inferior Parathyroid Glands

Pattern Type 0 - Parathyroid pedicle making no contact with the thyroid
Right Side - Inferior Parathyroid Glands

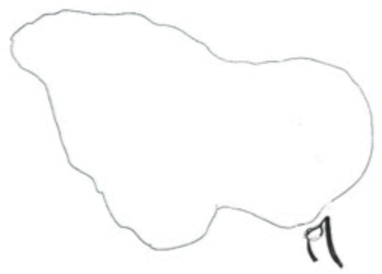

RI-0.1 iMAP2

Pattern Type 0 - Parathyroid pedicle making no contact with the thyroid
Left Side- Inferior Parathyroid Glands

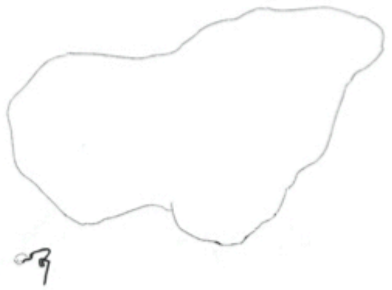

LI-0.1 iMAP2

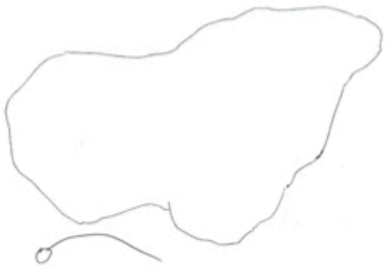

LI-0.2 iMAP2

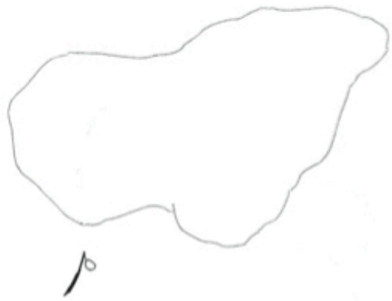

LI-0.3 iMAP2

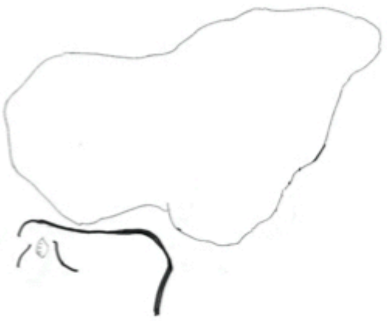

LI-0.4 iMAP1

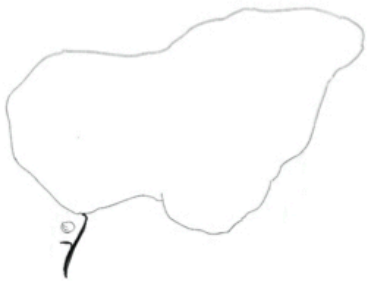

LI-0.5 *iMAP1*

Pattern Type 1 - Parathyroid pedicle making punctiform contact with the thyroid
Right Side - Inferior Parathyroid Glands

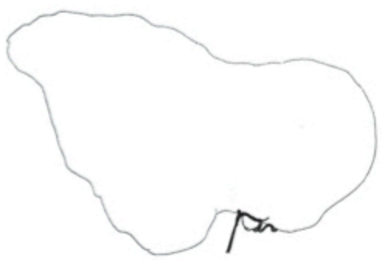

RI-1.1 *iMAP2*

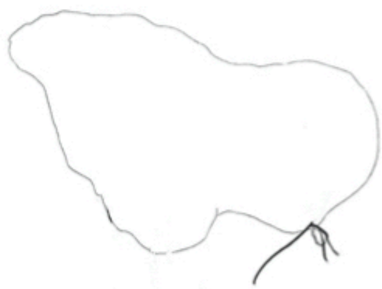

RI-1.2 *iMAP2*

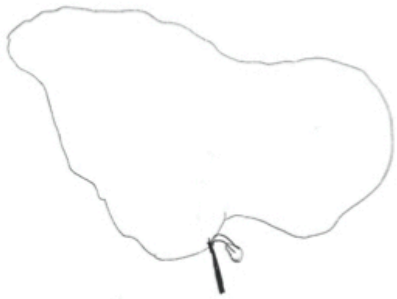

RI-1.3 *iMAP2*

Pattern Type 1 - Parathyroid pedicle making punctiform contact with the thyroid
Left Side - Inferior Parathyroid Glands

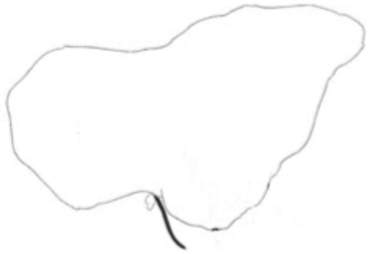

LI-1.1 iMAP1

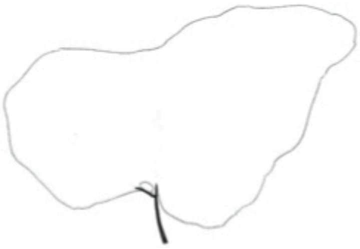

LI-1.2 iMAP2

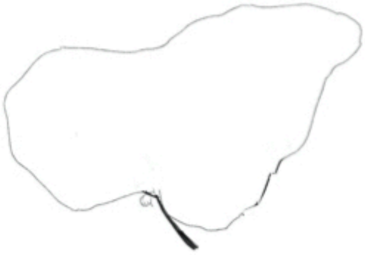

LI-1.3 iMAP2

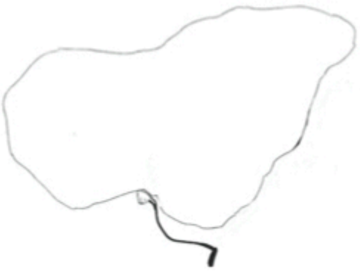

LI-1.4 iMAP2

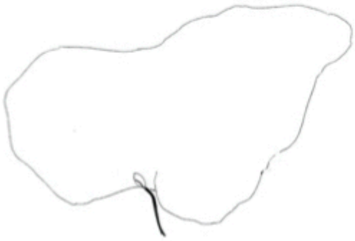

LI-1.5 iMAP1

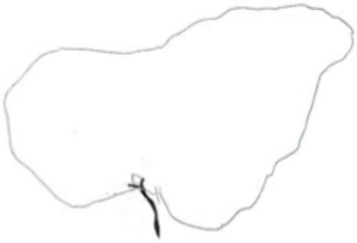

LI-1.6 iMAP2

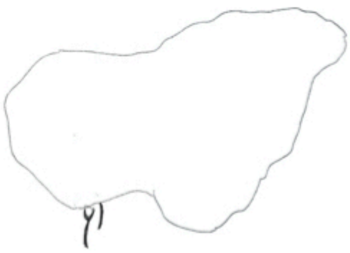

LI-1.7 iMAP2

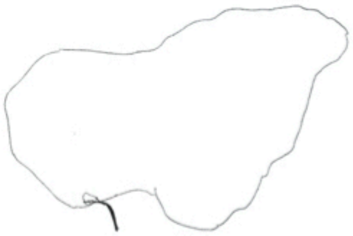

LI-1.8 iMAP1

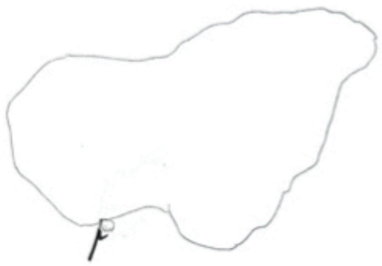

LI-1.9 *iMAP1*

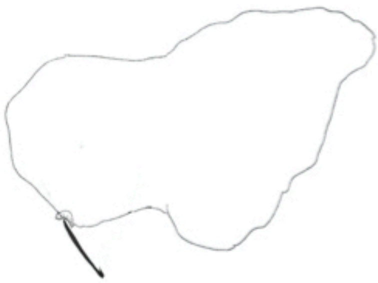

LI-1.10 *iMAP2*

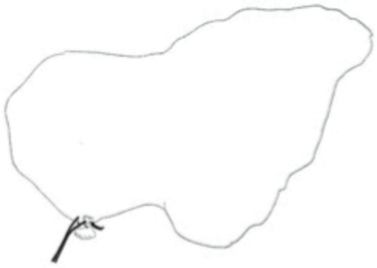

LI-1.11 *iMAP2*

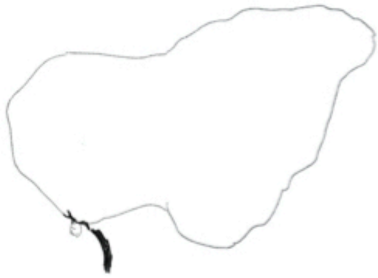

LI-1.12 *iMAP2*

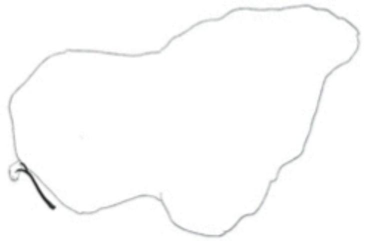

LI-1.13 *iMAP2*

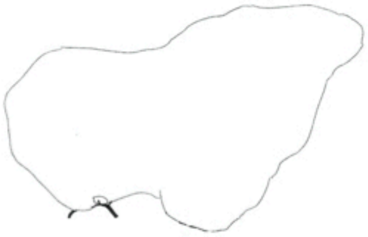

LI-1.14 *iMAP2*

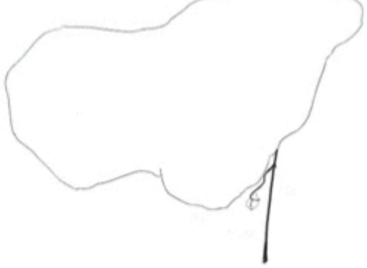

LI-1.15 *iMAP2*

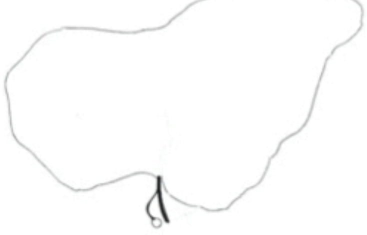

LI-1.16 *iMAP2*

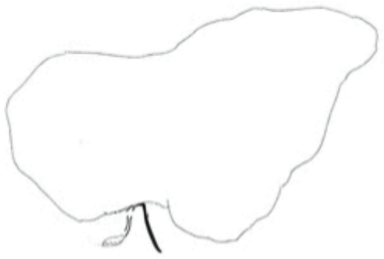

LI-1.17 iMAP1

Type 2 - Parathyroid pedicle running along the posterior edge of the thyroid
Right Side - Inferior Parathyroid Glands

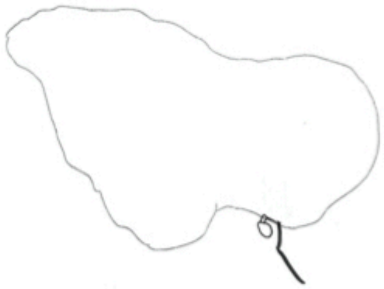

RI-2.1 iMAP2

Type 2 - Parathyroid pedicle running along the posterior edge of the thyroid
Left Side - Inferior Parathyroid Glands

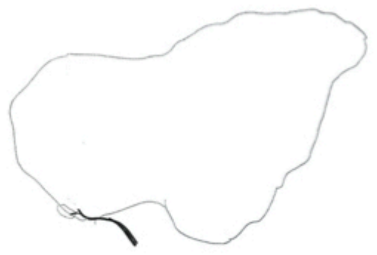

LI-2.1 iMAP2

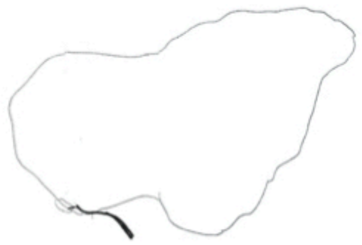

LI-2.2 iMAP2

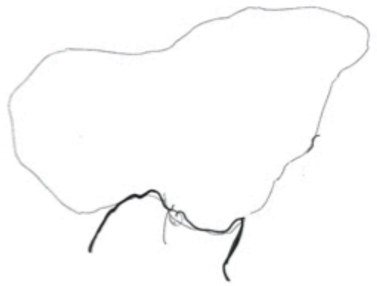

LI-2.3 iMAP1

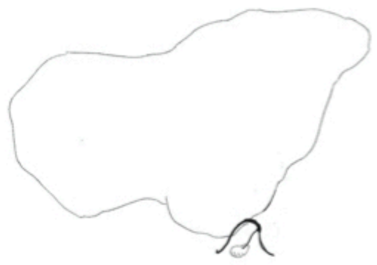

LI-2.4 iMAP2

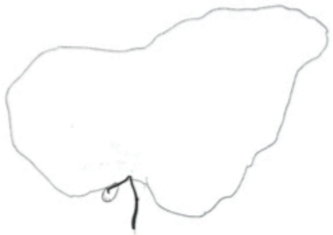

LI-2.5 iMAP2

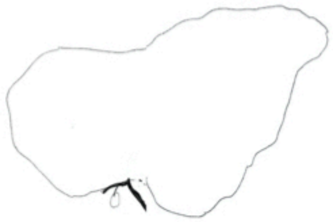

LI-2.6 iMAP2

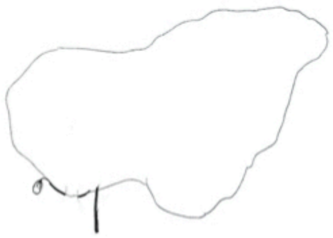

LI-2.7 iMAP1

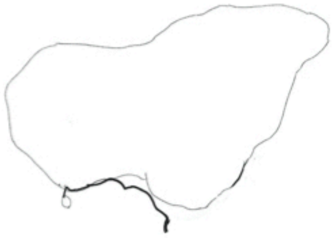

LI-2.8 iMAP2

Type 3 - Parathyroid pedicle running on the lateral face of the thyroid
Right Side - Inferior Parathyroid Glands

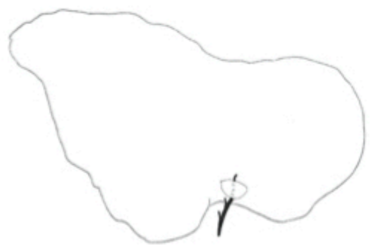

RI-3.1 iMAP2

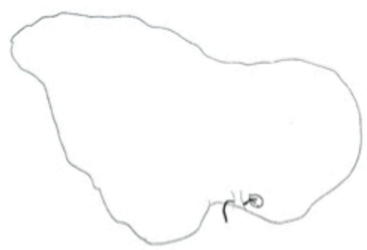

RI-3.2 iMAP2

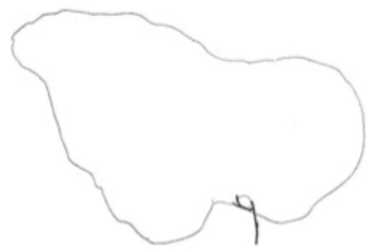

RI-3.3 iMAP1

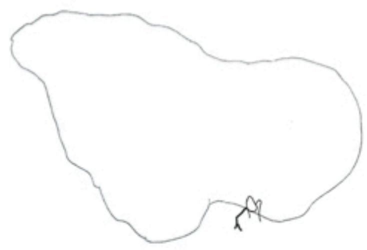

RI-3.4 iMAP1

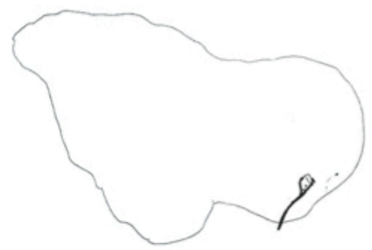

RI-3.5 iMAP2

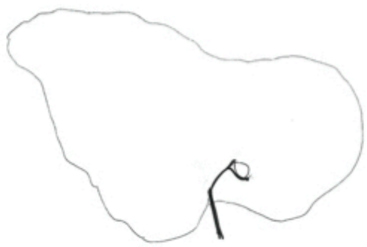

RI-3.6 iMAP2

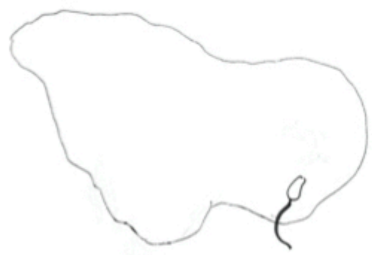

RI-3.7 iMAP2

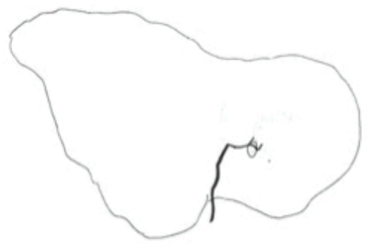

RI-3.8 iMAP2

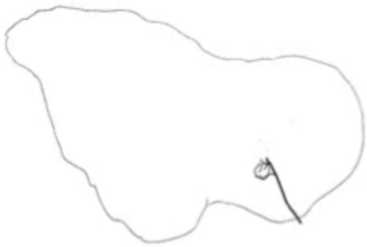

RI-3.9 *iMAP2*

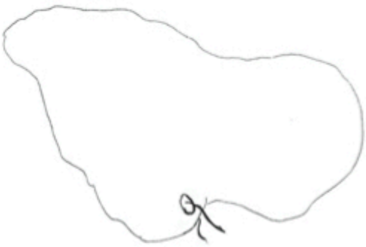

RI-3.10 *iMAP2*

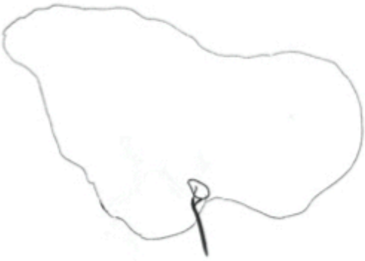

RI-3.11 *iMAP2*

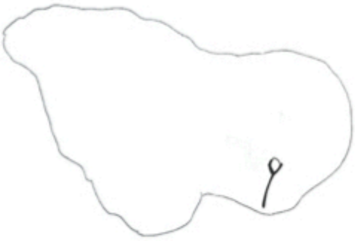

RI-3.12 *iMAP2*

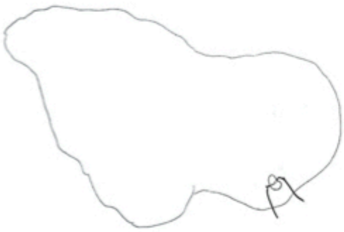

RI-3.13 *iMAP1*

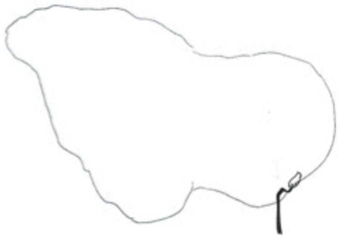

RI-3.14 *iMAP2*

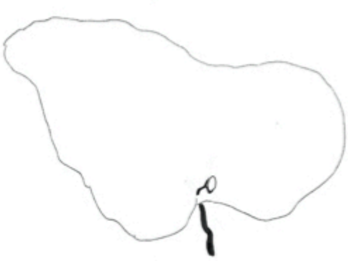

RI-3.15 *iMAP1*

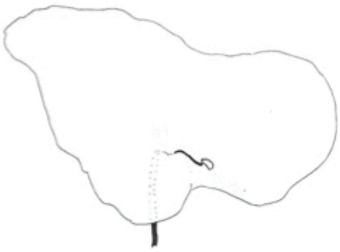

RI-3.16 *iMAP1*

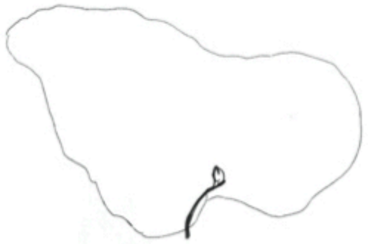

RI-3.17 iMAP2

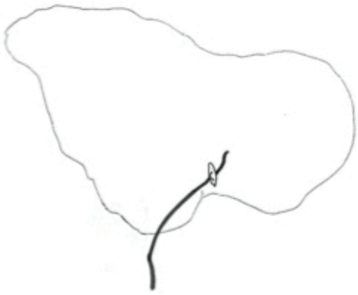

RI-3.18 iMAP2

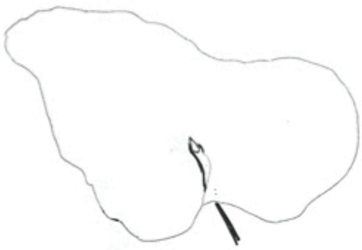

RI-3.19 iMAP2

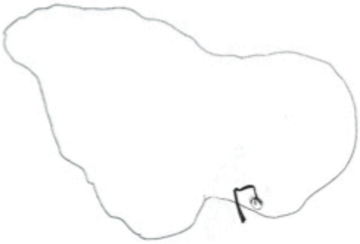

RI-3.20 iMAP2

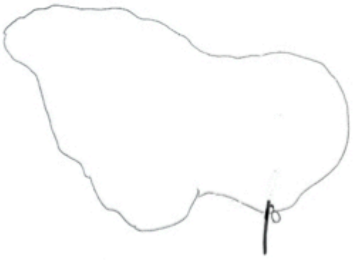

RI-3.21 iMAP1

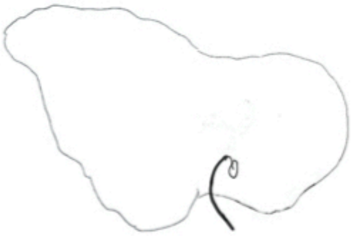

RI-3.22 iMAP2

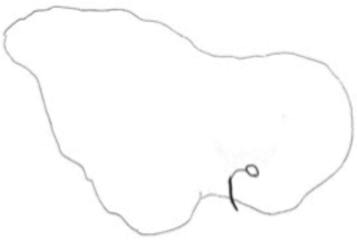

RI-3.23 iMAP2

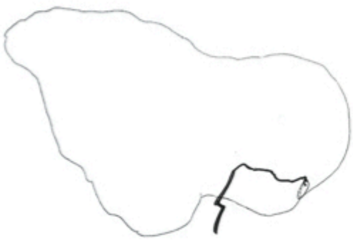

RI-3.24 iMAP2

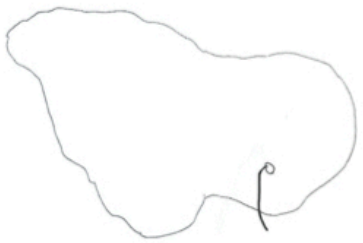

RI-3.25 *iMAP2*

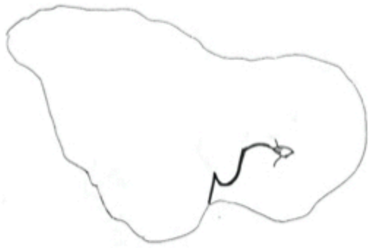

RI-3.26 *iMAP2*

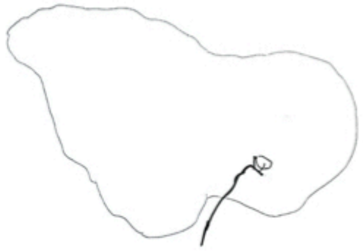

RI-3.27 *iMAP2*

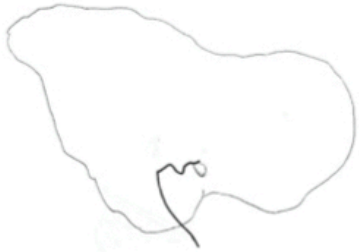

RI-3.28 *iMAP2*

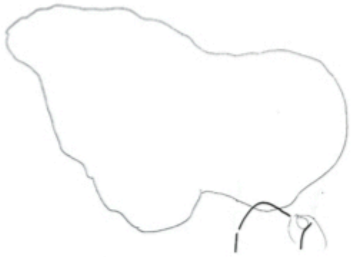

RI-3.29 *iMAP1*

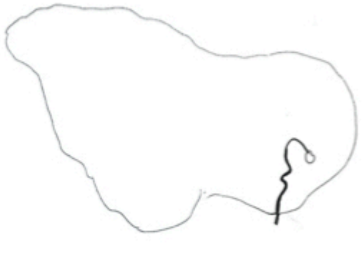

RI-3.30 *iMAP2*

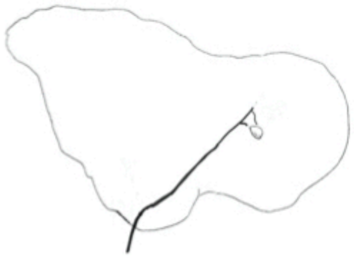

RI-3.31 *iMAP1*

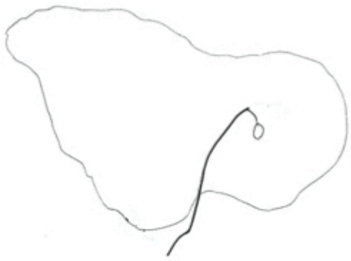

RI-3.32 *iMAP2*

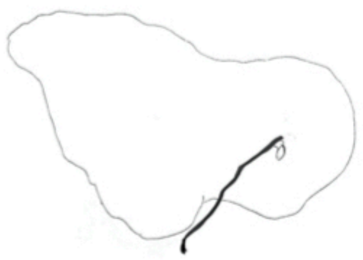

RI-3.33 *iMAP1*

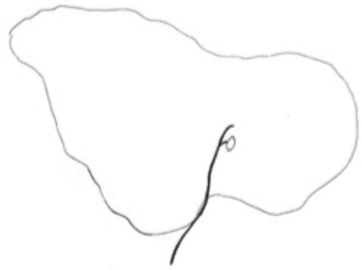

RI-3.34 *iMAP2*

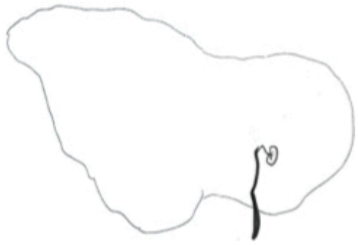

RI-3.35 *iMAP2*

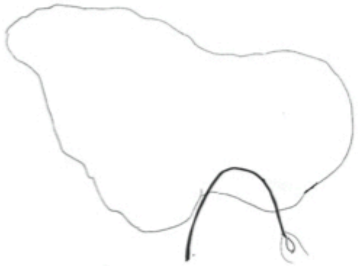

RI-3.36 *iMAP2*

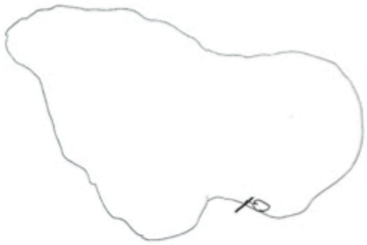

RI-3.37 *iMAP1*

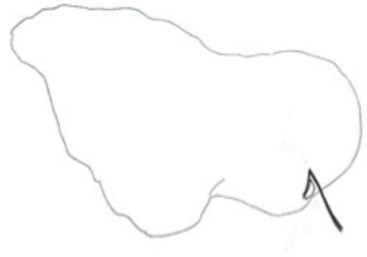

RI-3.38 *iMAP1*

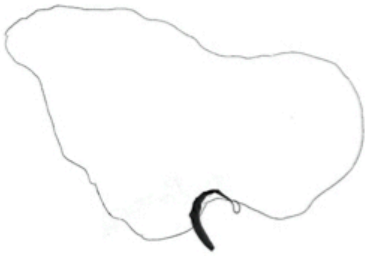

RI-3.39 *iMAP2*

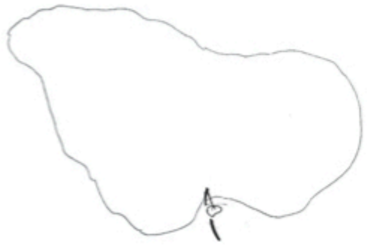

RI-3.40 *iMAP2*

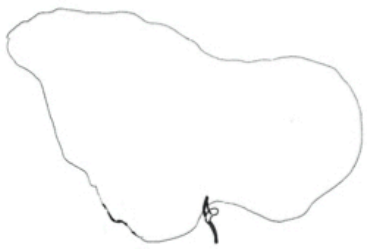

RI-3.41 iMAP2

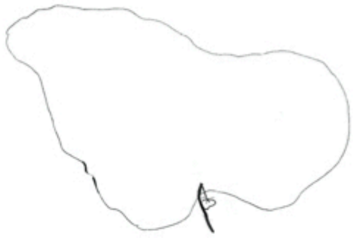

RI-3.42 iMAP2

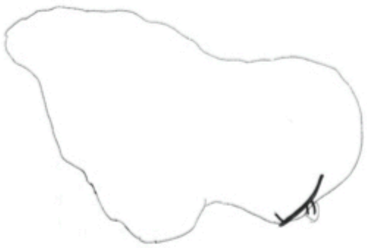

RI-3.43 iMAP1

Type 3 - Parathyroid pedicle running on the lateral face of the thyroid
Left Side - Inferior Parathyroid Glands

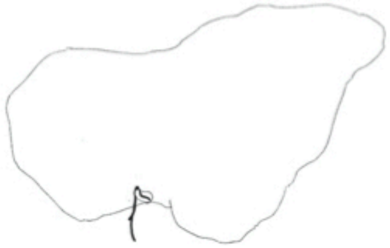

LI-3.1

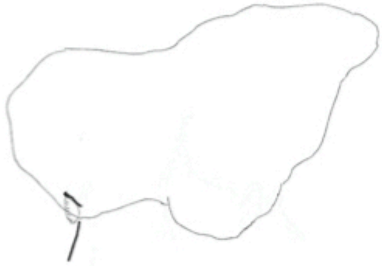

LI-3.2 iMAP1

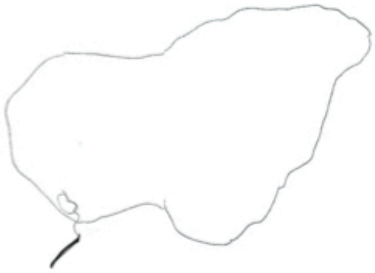

LI-3.3 iMAP0

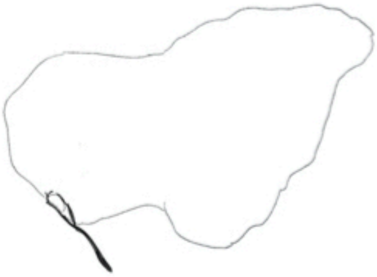

LI-3.4 iMAP2

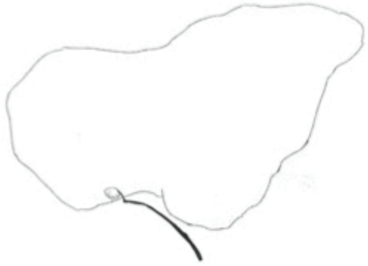

LI-3.5 *iMAP2*

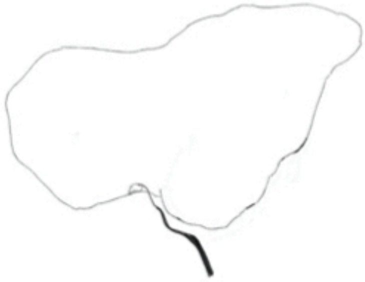

LI-3.6 *iMAP2*

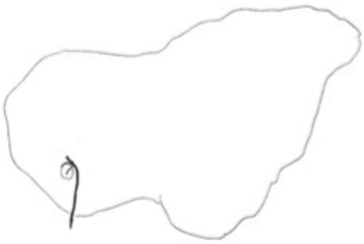

LI-3.7 *iMAP2*

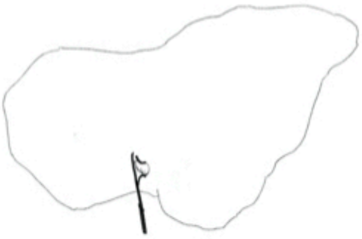

LI-3.8 *iMAP2*

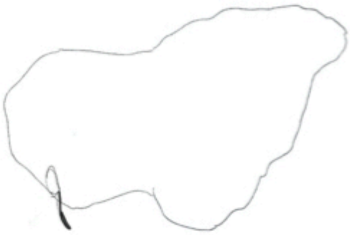

LI-3.9 *iMAP1*

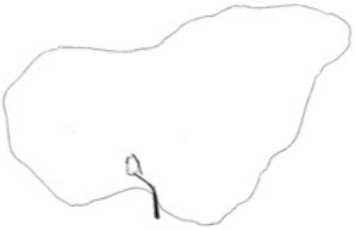

LI-3.10 *iMAP2*

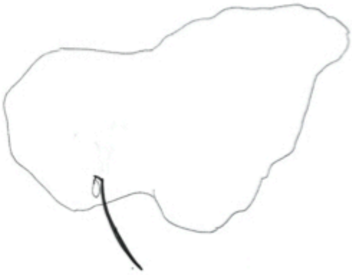

LI-3.11 *iMAP2*

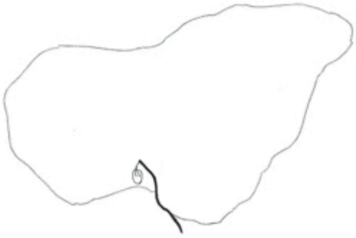

LI-3.12 *iMAP2*

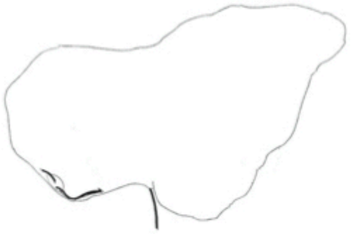

LI-3.13 *iMAP1*

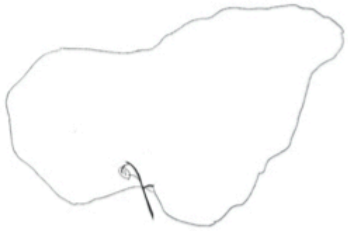

LI-3.14 *iMAP2*

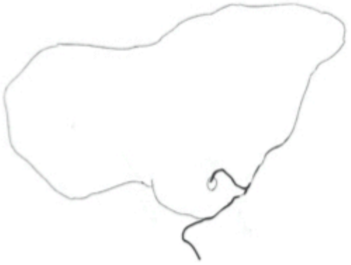

LI-3.15 *iMAP2*

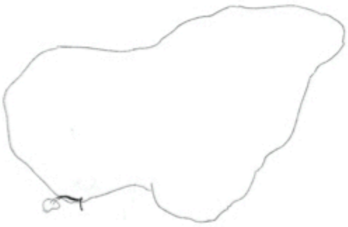

LI-3.16 *iMAP1*

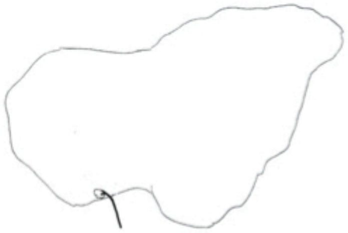

LI-3.17 *iMAP1*

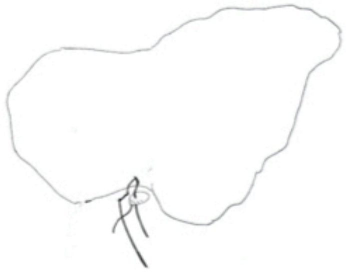

LI-3.18 *iMAP1*

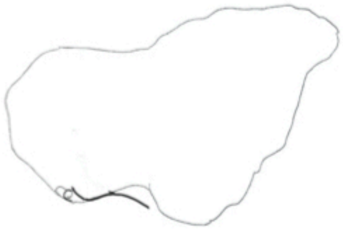

LI-3.19 *iMAP1*

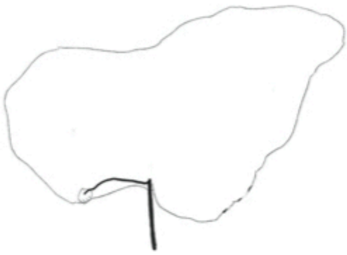

LI-3.20 *iMAP2*

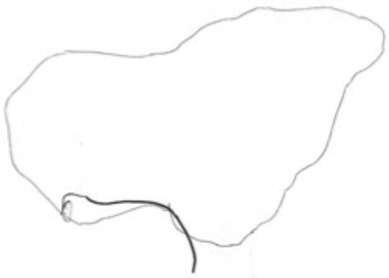

LI-3.21 *iMAP2*

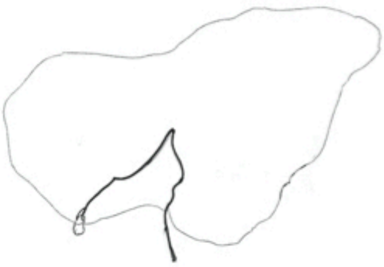

LI-3.22 *iMAP2*

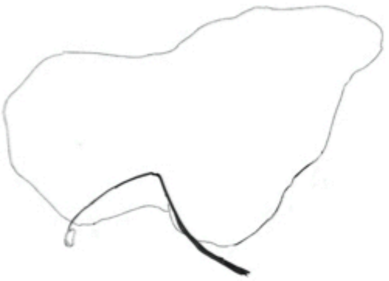

LI-3.23 *iMAP2*

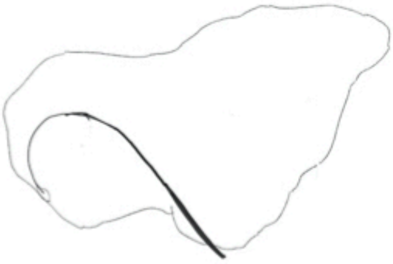

LI-3.24 *iMAP2*

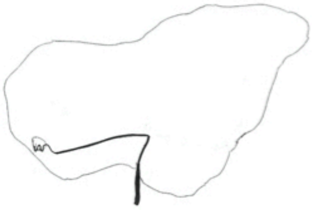

LI-3.25 *iMAP2*

Type 4 - Intrathyroid parathyroid
Right Side - Inferior Parathyroid Glands

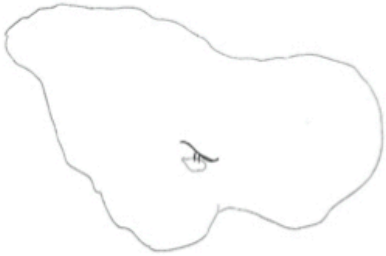

RI-4.1 iMAP0

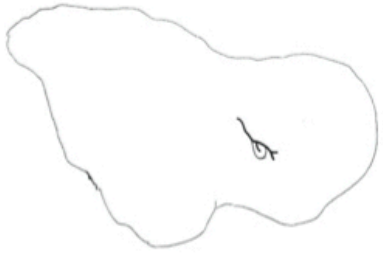

RI-4.2 iMAP0

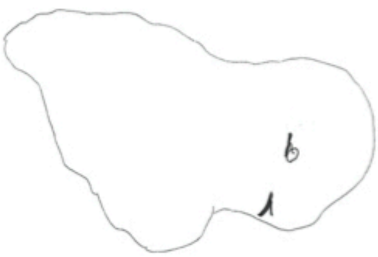

RI-4.3 iMAP0

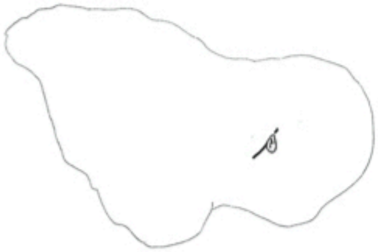

RI-4.4 iMAP0

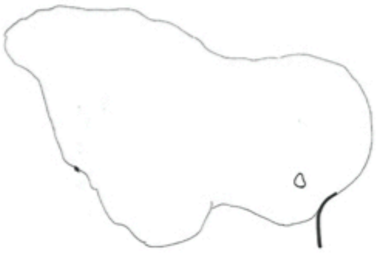

RI-4.5 iMAP0

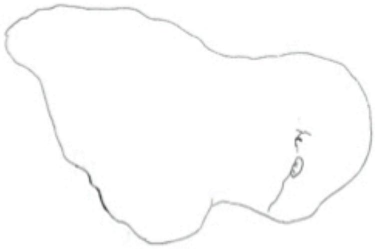

RI-4.6 iMAP0

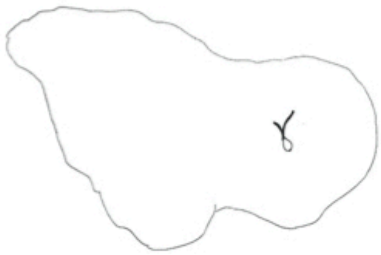

RI-4.7 iMAP0

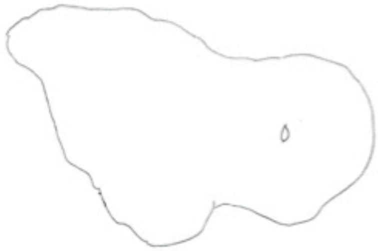

RI-4.8 iMAP0

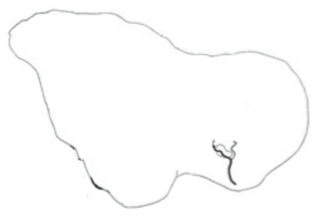

RI-4.9 *iMAP0*

Type 4 - Intrathyroid parathyroid
Left Side - Inferior Parathyroid Glands

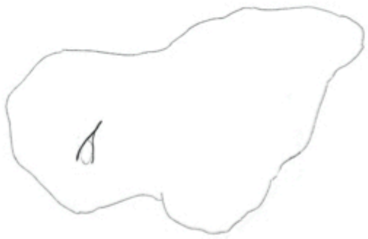

LI-4.1 *iMAP0*

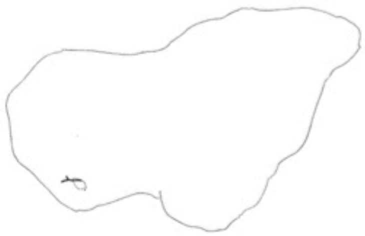

LI-4.2 *iMAP0*

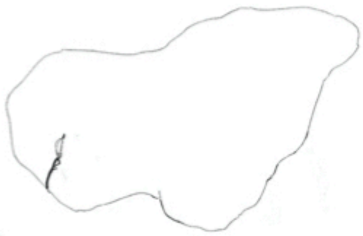

LI-4.3 *iMAP1*

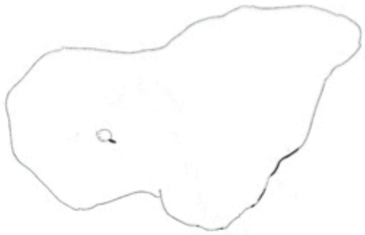

LI-4.4 *iMAP0*

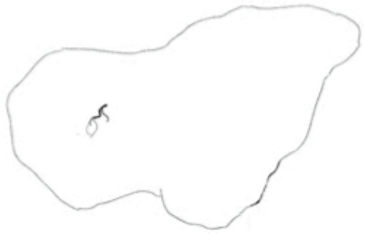

LI-4.5 *iMAP0*

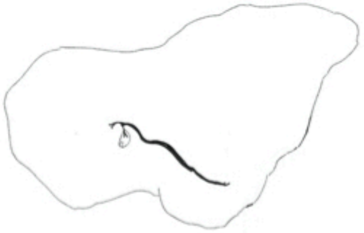

LI-4.6 *iMAP1*

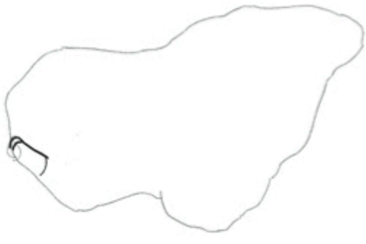

LI-4.7 *iMAP0*

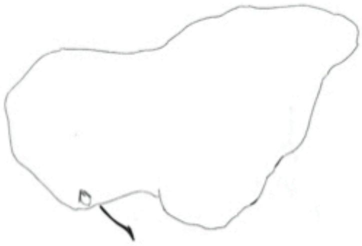

LI-4.8 *iMAP1*

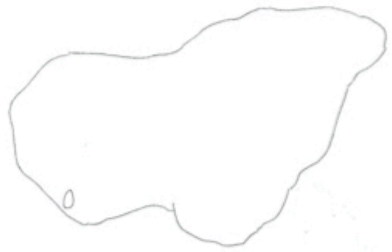

LI-4.9 *iMAP0*

Type X1 - Parathyroid pedicle possibly running on the medial face of the thyroid
Right Side - Inferior Parathyroid Glands

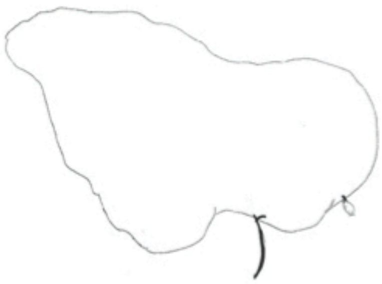

RI-X1.1 *iMAP1*

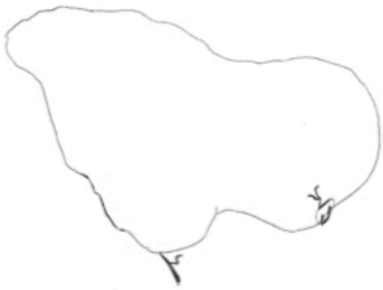

RI-X1.2 *iMAP0*

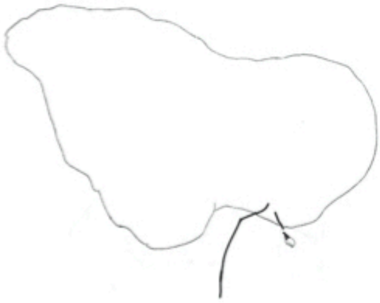

RI-X1.3 *iMAP1*

Type X1 - Parathyroid pedicle possibly running on the medial face of the thyroid
Left Side - Inferior Parathyroid Glands

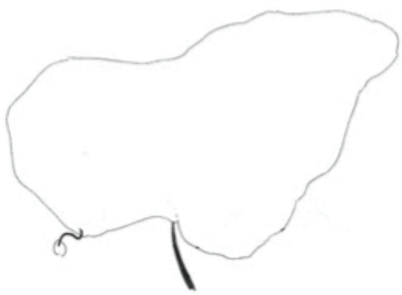

LI-X1.1 iMAP1

Type X2 - Parathyroid pedicle path unknown
Right Side - Superior Parathyroid Glands

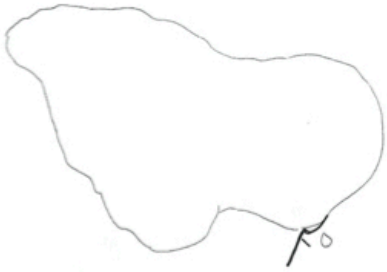

RI-X2.1 iMAP0

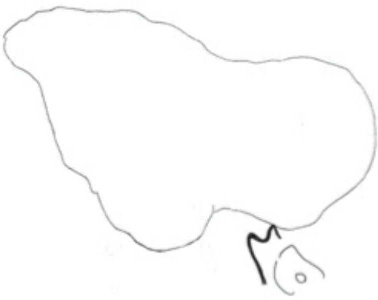

RI-X2.2 iMAP1

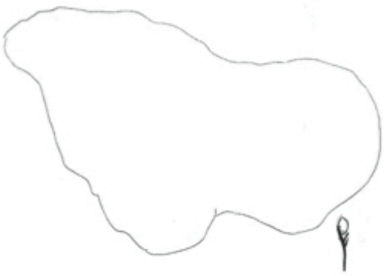

RI-X2.3 iMAP1

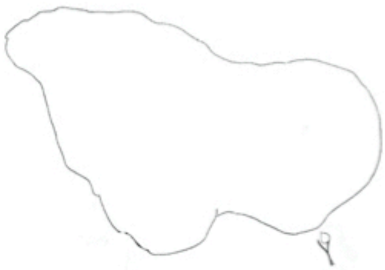

RI-X2.4 iMAP1

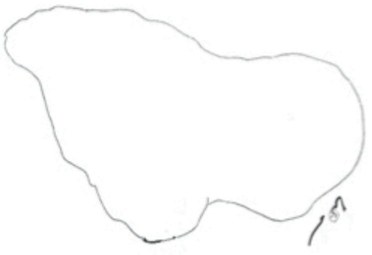

RI-X2.5 iMAP1

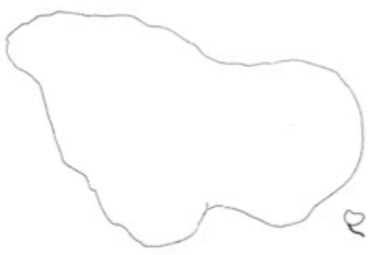

RI-X2.6 iMAP0

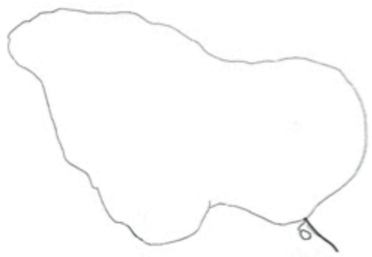

RI-X2.7 iMAP1

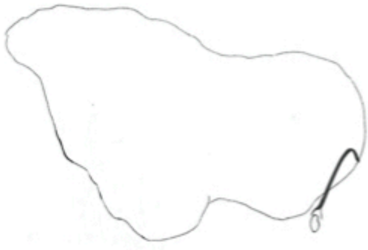

RI-X2.8 iMAP1

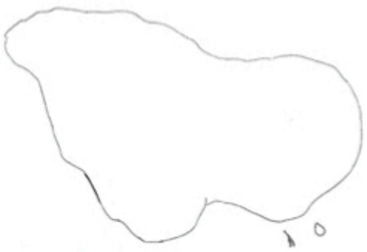

RI-X2.9 iMAP0

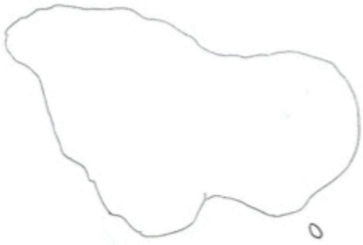

RI-X2.10 iMAP0

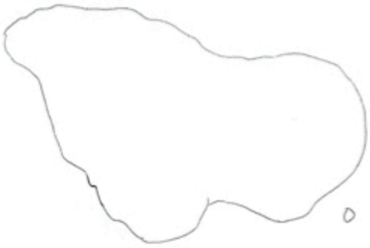

RI-X2.11 iMAP0

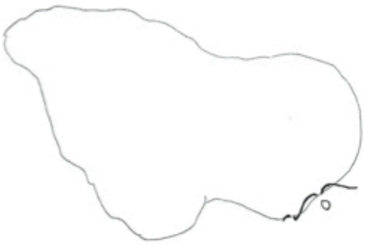

RI-X2.12 iMAP1

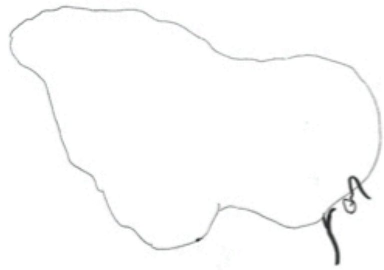

RI-X2.13 iMAP1

Type X2 - Parathyroid pedicle path unknown
Left Side - Superior Parathyroid Glands

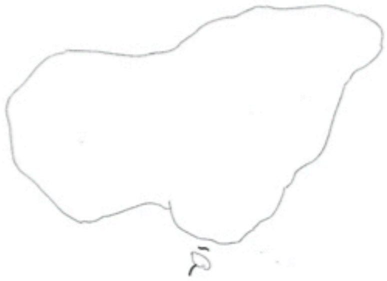

LI-X2.1 iMAP0

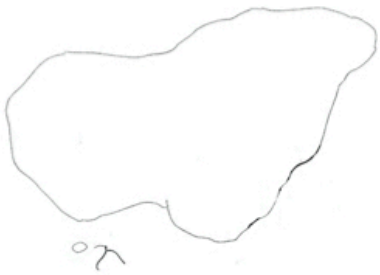

LI-X2.2 iMAP1

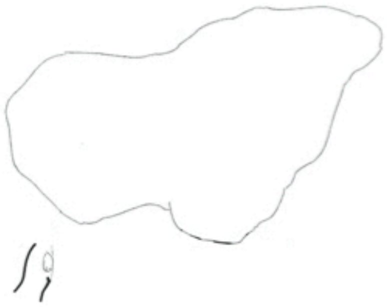

LI-X2.3 iMAP1

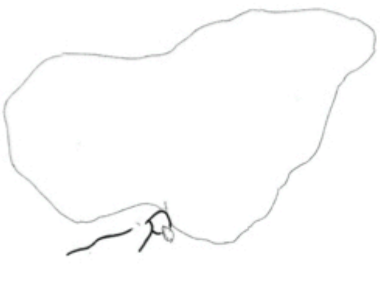

LI-X2.4 iMAP1

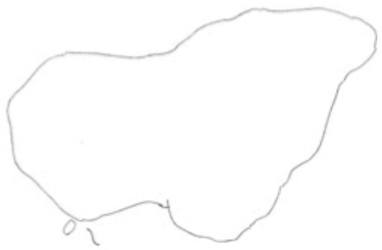

LI-X2.5 *iMAP0*

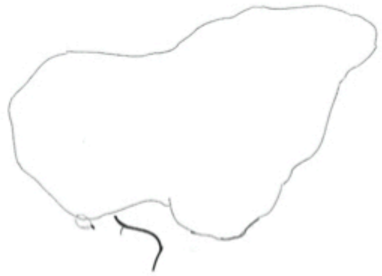

LI-X2.6 *iMAP1*

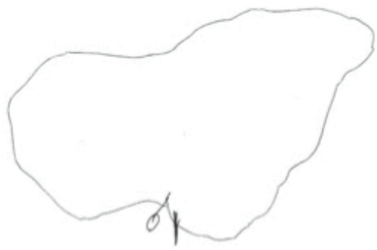

LI-X2.7 *iMAP1*

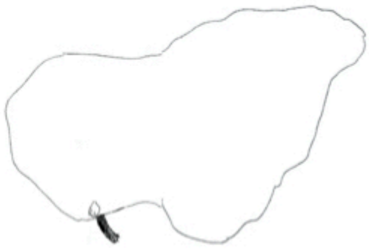

LI-X2.8 *iMAP1*

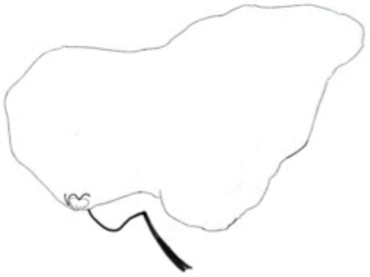

LI-X2.9 *iMAP1*

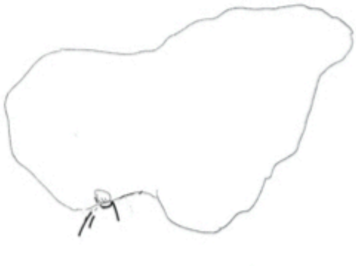

LI-X2.10 *iMAP1*
