# Supplementary material for: Parathyroid vascular anatomy using intraoperative mapping angiography: the PARATLAS study
Source: Br J Surg. 2025 Mar 11;112(3):znae307. doi: 10.1093/bjs/znae307 (PMC11894526; doi:10.1093/bjs/znae307)
Supplement: znae307_Supplementary_Data [file znae307_supplementary_data.zip › Supplementary_material.docx]

**Supplementary material**

Parathyroid vascular anatomy using intraoperative mapping angiography: the PARATLAS study

Fares Benmiloud ^1^, Neil Tolley ^2^, Anne Denizot ^1^, Aimee MA Di Marco ^2,3^, Frederic Triponez ^4^

^1^ Hôpital Européen Marseille, Marseille, France

^2^ Imperial College Healthcare NHS Trust, London, United Kingdom

^3^ Imperial College Healthcare NHS Trust, Endocrine Surgery Unit, London, United Kingdom

^4^University Hospitals of Geneva, Geneva, Switzerland

**Corresponding author:**

Fares Benmiloud, Hôpital Européen Marseille, 6 rue Désirée Clary, 13003, Marseille, France. [faresbenmiloud@hotmail.com](mailto:faresbenmiloud@hotmail.com) (email address and ORCID ID)

**Supplementary Materials - Index**

| **Supplementary Figures and Tables** |  |
| --- | --- |
| Table S1. Correlation between parathyroid location and angiography results | *pag. 2* |
| **Supplementary Appendixes** |  |
| Comprehensive and systematic atlas of the path of the parathyroid vessels in relation to the thyroid, based on intraoperative angiographies performed on 200 patients. | *See attached file* |

**Supplementary Table**

**Table S1. Correlation between parathyroid location and angiography results**

|  | **(A) Right superior PG** | **(B)**  **Left superior PG** | **(C)**  **Right inferior PG** | **(D)**  **Left inferior PG** | **A vs. B**  **p value** | **C vs. D**  **p value** | **(A+B) vs. (C+D)**  **p value** |
| --- | --- | --- | --- | --- | --- | --- | --- |
| **iMAP 2** | 53 (65%) | 56  (63%) | 36 (49%) | 39  (51%) | 0.8994 | 0.3222 | 0.0459* |
| **iMAP 1** | 19 (23%) | 20  (23%) | 22 (30%) | 28  (37%) |  |  |  |
| **iMAP 0** | 10 (12%) | 13  (15%) | 15 (21%) | 9  (12%) |  |  |  |
| **Total** | 82 | 89 | 73 | 76 |  |  |  |

*p=0.0459 indicates a significant difference between superior and inferior for at least one iMAP Type. After testing two-by-two multiple comparisons (Tukey style test for multiple comparisons) no significant difference between superior and inferior was confirmed; an effect exists but the data do not allow any formal conclusions.
